# Evidence‐informed practice versus evidence‐based practice educational interventions for improving knowledge, attitudes, understanding, and behavior toward the application of evidence into practice: A comprehensive systematic review of UG student

**DOI:** 10.1002/cl2.1233

**Published:** 2022-04-16

**Authors:** Elizabeth A. Kumah, Robert McSherry, Josette Bettany‐Saltikov, Paul van Schaik, Sharon Hamilton, Julie Hogg, Vicki Whittaker

**Affiliations:** ^1^ Faculty of Health and Social Care University of Chester Chester UK; ^2^ School of Health and Life Sciences Teesside University Middlesbrough UK; ^3^ School of Social Sciences, Humanities and Law Teesside University Middlesbrough UK

## Abstract

**Background:**

To produce graduates with strong knowledge and skills in the application of evidence into healthcare practice, it is imperative that all undergraduate health and social care students are taught, in an efficient manner, the processes involved in applying evidence into practice. The two main concepts that are linked to the application of evidence into practice are “evidence‐based practice” and “evidence‐informed practice.” Globally, evidence‐based practice is regarded as the gold standard for the provision of safe and effective healthcare. Despite the extensive awareness of evidence‐based practice, healthcare practitioners continue to encounter difficulties in its implementation. This has generated an ongoing international debate as to whether evidence‐based practice should be replaced with evidence‐informed practice, and which of the two concepts better facilitate the effective and consistent application of evidence into healthcare practice.

**Objectives:**

The primary objective of this systematic review was to evaluate and synthesize literature on the effectiveness of evidence‐informed practice versus evidence‐based practice educational interventions for improving knowledge, attitudes, understanding, and behavior of undergraduate health and social care students toward the application of evidence into practice. Specifically, we planned to answer the following research questions: (1) Is there a difference (i.e., difference in content, outcome) between evidence‐informed practice and evidence‐based practice educational interventions? (2) Does participating in evidence‐informed practice educational interventions relative to evidence‐based practice educational interventions facilitate the application of evidence into practice (as measured by, e.g., self‐reports on effective application of evidence into practice)? (3) Do both evidence‐informed practice and evidence‐based practice educational interventions targeted at undergraduate health and social care students influence patient outcomes (as measured by, e.g., reduced morbidity and mortality, absence of nosocomial infections)? (4) What factors affect the impact of evidence‐informed practice and evidence‐based practice educational interventions (as measured by, e.g., course content, mode of delivery, multifaceted interventions, standalone intervention)?

**Search Methods:**

We utilized a number of search strategies to identify published and unpublished studies: (1) Electronic databases: we searched Academic Search Complete, Academic search premier, AMED, Australian education index, British education index, Campbell systematic reviews, Canada bibliographic database (CBCA Education), CINAHL, Cochrane Library, Database of Abstracts of Reviews on Effectiveness, Dissertation Abstracts International, Education Abstracts, Education complete, Education full text: Wilson, ERIC, Evidence‐based program database, JBI database of systematic reviews, Medline, PsycInfo, Pubmed, SciELO (Scientific Electronic Library Online), and Scopus; (2) A web search using search engines such as Google and Google scholar; (3) Grey literature search: we searched OpenGrey (System for Information on Grey Literature in Europe), System for information on Grey Literature, the Society for Research on Educational Effectiveness, and Virginia Henderson Global Nursing e‐Repository; (4) Hand searching of journal articles; and (5) Tracking bibliographies of previously retrieved studies. The searches were conducted in June 2019.

**Selection Criteria:**

We planned to include both quantitative (including randomized controlled trials, non‐randomized controlled trials, quasi‐experimental, before and after studies, prospective and retrospective cohort studies) and qualitative primary studies (including, case series, individual case reports, and descriptive cross‐sectional studies, focus groups, and interviews, ethnography, phenomenology, and grounded theory), that evaluate and compare the effectiveness of any formal evidence‐informed practice educational intervention to evidence‐based practice educational intervention. The primary outcomes were evidence‐informed practice and evidence‐based practice knowledge, attitudes, understanding, and behavior. We planned to include, as participants, undergraduate pre‐registration health and social care students from any geographical area.

**Data Collection and Analysis:**

Two authors independently screened the search results to assess articles for their eligibility for inclusion. The screening involved an initial screening of the title and abstracts, and subsequently, the full‐text of selected articles. Discrepancies were resolved through discussion or consultation with a third author. We found no article eligible for inclusion in this review.

**Main Results:**

No studies were found which were eligible for inclusion in this review. We evaluated and excluded 46 full‐text articles. This is because none of the 46 studies had evaluated and compared the effectiveness of evidence‐informed practice educational interventions with evidence‐based practice educational interventions. Out of the 46 articles, 45 had evaluated solely, the effectiveness of evidence‐based practice educational interventions and 1 article was on evidence‐informed practice educational intervention. Hence, these articles were excluded as they did not meet the inclusion criteria.

**Authors' Conclusions:**

There is an urgent need for primary studies evaluating the relative effectiveness of evidence‐informed practice and evidence‐based practice educational interventions targeted at improving undergraduate healthcare students' competencies regarding the application of evidence into practice. Such studies should be informed by current literature on the concepts (i.e., evidence‐informed practice and evidence‐based practice) to identify the differences, similarities, as well as appropriate content of the educational interventions. In this way, the actual effect of each of the concepts could be determined and their effectiveness compared.

## 1 PLAIN LANGUAGE SUMMARY

### 1.1 Evidence‐informed versus evidence‐based practice educational interventions for improving knowledge, attitudes, understanding, and behavior toward the application of evidence into practice: A comprehensive systematic review of undergraduate students

We found no studies that compared the effectiveness of evidence‐informed practice educational interventions to evidence‐based practice educational interventions in targeting undergraduate health and social care students' knowledge, attitudes, understanding, and behavior.

### 1.2 The review in brief

This review aimed to compare the relative effectiveness of evidence‐informed practice versus evidence‐based practice educational interventions on the knowledge, attitudes, understanding, and behavior of undergraduate health and social care students. We did not find any studies that met our inclusion criteria, therefore we cannot draw any conclusions regarding the relative effectiveness of the two approaches. The evidence is current to June 17, 2019.

### 1.3 What is this review about?

The effective application of the best evidence into healthcare practice is strongly endorsed, alongside a growing need for healthcare organizations to ensure the delivery of services in an equitable and efficient manner. Existing evidence shows that guiding healthcare practice with the best available evidence enhances healthcare delivery, improves efficiency and ultimately improves patient outcomes. Nevertheless, there is often the ineffective and inconsistent application of evidence into healthcare practice.

The two main concepts that have been associated with the application of evidence into healthcare practice are “evidence‐based practice” and “evidence‐informed practice.” This review assesses the relative effectiveness of these two approaches, specifically in relation to improving knowledge, attitudes, understanding, and behavior of undergraduate health and social care students. In addition, we aimed to assess the impact of evidence‐informed practice and/or evidence‐based practice educational programmes on patient outcomes. Examples of patient outcome indicators that we would have assessed had eligible studies been found are: user experience, length of hospital stay, nosocomial infections, patient and health practitioner satisfaction, mortality, and morbidity rates.

**What is the aim of this review?**

This Campbell systematic review examines the effectiveness of evidence‐informed practice and evidence‐based practice educational interventions for improving knowledge, attitudes, understanding, and behavior of undergraduate health and social care students toward the application of evidence into practice.

### 1.4 What studies are included?

We planned to include both quantitative and qualitative studies aimed at improving knowledge, attitudes, understanding, and behavior of undergraduate pre‐registration health and social care students from any geographical area. Studies whose participants were registered health and social care practitioners and postgraduate students were excluded.

We planned to include studies that were published between 1996 and June 2019. No limit was placed on the language of publication.

### 1.5 What are the main findings of this review?

A total of 45 full‐text articles on evidence‐based practice educational interventions and one full‐text article on evidence‐informed practice educational intervention were screened for their eligibility for inclusion. However, we identified no studies examining the relative effectiveness of evidence‐informed practice versus evidence‐based practice educational interventions. As a result, we are unable to answer the question as to which of the two concepts better facilitates the application of evidence into healthcare practice.

### 1.6 What do the findings of this review mean?

Whilst evidence suggests that evidence‐informed practice can be effective (compared to a no‐intervention control) in improving student outcomes, we are unable to conclude which approach better facilitates the application of evidence into practice.

### 1.7 How up‐to‐date is this review?

The review authors searched for studies published up to June 2019.

## 2 BACKGROUND

### 2.1 Description of the condition

Over the past three decades, there has been increasing attention on improving healthcare quality, reliability, and ultimately, patient outcomes, through the provision of healthcare that is influenced by the best available evidence, and devoid of rituals and tradition (Andre et al., 2016; Melnyk et al., 2014; Sackett et al., 1996). There is an expectation by professional regulators such as the Nursing and Midwifery Council, United Kingdom (Nursing and Midwifery Council, 2015) and the Health and Care Professions Council (Health and Care Professions Council, 2012) that the professional, as part of their accountability applies the best available evidence to inform their clinical decision‐making, roles, and responsibilities. This is imperative for several reasons. First, it enhances the delivery of healthcare and improves efficiency. Second, it produces better intervention outcomes and promotes transparency. Third, it enhances co‐operation and knowledge sharing among professionals and service users, and ultimately, the effective application of evidence into practice improves patient outcomes and enhances job satisfaction. Indeed, the need to guide healthcare practice with evidence has been emphasized by several authors, including Kelly et al. (2015), Nevo and Slonim‐Nevo (2011), Scott and Mcsherry (2009), Shlonsky and Stern (2007), Smith and Rennie (2014) Straus et al. (2011), Tickle‐Degnen and Bedell (2003), and Sackett et al. (1996). According to these authors, the effective and consistent application of evidence into practice helps practitioners to deliver the best care to their patients and patient relatives.

Two main concepts have been associated with the application of evidence into healthcare practice: “evidence‐based practice” and “evidence‐informed practice.” Evidence‐based practice is an offshoot of evidence‐based medicine; hence, the universally accepted definition of evidence‐based practice is adapted from the definition of evidence‐based medicine, which is “the conscientious, explicit and judicious use of the best evidence in making decisions about the care of the individual patient” (Sackett et al., 1996, p. 71). Evidence‐informed practice, on the other hand, is defined as the assimilation of professional judgment and research evidence regarding the efficiency of interventions (McSherry et al., 2002). This definition was further elaborated by Nevo and Slonim‐Nevo, 2011 as an approach to patient care where:

Practitioners are encouraged to be knowledgeable about findings coming from all types of studies and to use them in an integrative manner, taking into consideration clinical experience and judgment, clients' preferences and values, and context of the interventions (p. 18).

The primary aim of both evidence‐informed practice and evidence‐based practice is to facilitate the application of evidence into healthcare practice. However, there are significant differences between the two concepts. These differences are discussed in detail in the ensuing sections. Nonetheless, it is important to note here that a characteristic difference between evidence‐informed practice and evidence‐based practice is the processes involved in applying the concepts. Evidence‐based practice provides a step‐wise approach to the application of evidence into practice, where practitioners are required to follow a series of steps to implement evidence‐based practice. According to Sackett (2000), the core steps of evidence‐based practice include: (1) formulating a clinical question, (2) searching the literature for the best research evidence to answer the question, (3) critically appraising the research evidence, (4) integrating the appraised evidence with own clinical expertise, patient preferences, and values, and (5) evaluating outcomes of decision‐making.

Evidence‐informed practice, on the other hand, offers an integrated, all‐inclusive approach to the application of evidence into practice (Nevo & Slonim‐Nevo, 2011). As illustrated by McSherry (2007), evidence‐informed practice provides a systems‐based approach (made up of input, throughput, and output) to applying evidence into practice, which contains, as part of its elements, the steps of evidence‐based practice. Besides, unlike evidence‐based practice, the main process involved in the implementation of evidence‐informed practice is cyclical and interdependent (McSherry et al., 2002).

Evidence‐based practice is a well‐established concept in health and social care (Titler, 2008) and is regarded as the norm for the delivery of efficient healthcare service. In recent times, however, the concept of evidence‐informed practice is often used instead of evidence‐based practice. For example, in countries such as Canada, the term has been widely adopted and is used more often in the health and social care fields. This was reflected in a position statement by the Canadian Nurses Association (CNA, 2008) and the Canadian Physiotherapy Association (Canadian Physiotherapy Association, 2017), where healthcare practitioners, including nurses, clinicians, researchers, educators, administrators, and policy‐makers were encouraged to collaborate with other stakeholders to enhance evidence‐informed practice, to ensure integration of the healthcare system. In the United Kingdom, the term evidence‐informed practice has been extensively adopted in the field of education, with a lot of resources being invested to assess the progress toward evidence‐informed teaching (Coldwell et al., 2017). In addition, an evidence‐informed chartered college of teaching has been launched (Bevins et al., 2011) to ensure evidence‐informed teaching and learning.

Whilst evidence‐based practice has been considered the gold standard for effective healthcare delivery, a large majority of healthcare practitioners continue to encounter multiple difficulties, which inhibit the rapid application of evidence into practice (Epstein, 2009; Glasziou, 2005; Greenhalgh et al., 2014; McSherry, 2007; McSherry et al., 2002; Melnyk, 2017; Nevo & Slonim‐Nevo, 2011). This has generated an on‐going international debate as to whether the term “evidence‐based practice” should be replaced by “evidence‐informed practice,” and which of the two concepts best facilitate the effective and consistent application of evidence into practice. Researchers, such as Melnyk (2017), Melnyk and Newhouse (2014), and Gambrill (2010) believe that knowledge and skills in evidence‐based practice help the healthcare professional to effectively apply evidence into practice. Conversely, Epstein (2009), Nevo and Slonim‐Nevo (2011), and McSherry (2007) have argued the need to equip healthcare professionals with the necessary knowledge and skills of evidence‐informed practice to facilitate the effective and consistent application of evidence into practice. According to Nevo and Slonim‐Nevo (2011), the application of evidence into practice should, in principle be “informed by” evidence and not necessarily “based on” evidence. This suggests that decision‐making in healthcare practice “might be enriched by prior research but not limited to it” (Epstein, 2009, p. 9).

It is imperative that healthcare training institutions produce graduates who are equipped with the knowledge and skills necessary for the effective and consistent application of evidence into practice (Dawes et al., 2005; Frenk et al., 2010; Melnyk, 2017). Hence, healthcare training institutions are required to integrate the principles and processes involved in the application of evidence into undergraduate health and social care curricula. However, the question that often arises is: which of the two concepts (i.e., evidence‐informed practice and evidence‐based practice) best facilitates the application of evidence into practice? While Melnyk et al. (2010) have suggested a seven‐step approach to the application of evidence into practice (termed the “evidence‐based practice model”), as stated earlier, McSherry (2007) has argued that the principle involved in the application of evidence into practice is a systems‐based approach, with an input, throughput and an output (named the “evidence‐informed practice model”).

The main purpose of this systematic review was to determine the differences and similarities, if any, between evidence‐informed practice and evidence‐based practice educational interventions; as well as explore the role each concept plays in the application of evidence into practice. In addition, the present review aimed at determining whether the two concepts act together, or individually to facilitate the effective application of evidence into practice. We hoped to achieve these aims by reviewing published and unpublished primary papers that have evaluated and compared the effectiveness of evidence‐informed practice educational interventions with evidence‐based practice educational interventions targetted at improving undergraduate pre‐registration health and social care students' knowledge, attitudes, understanding, and behavior regarding the application of evidence into practice.

### 2.2 Description of the intervention

The gap between evidence and healthcare practice is well acknowledged (Lau et al., 2014; Melnyk, 2017; Straus et al., 2009). Difficulties in using evidence to make decisions in healthcare practice are evident across all groups of decision‐makers, including health care providers, policymakers, managers, informal caregivers, patients, and patient relatives (Straus et al., 2009). Consequently, several interventions have been developed to improve the implementation of evidence into healthcare practice and policy. Specifically, evidence‐based practice educational interventions are widely used and have been greatly evaluated (e.g., Callister et al., 2005; Dawley et al., 2011; Heye & Stevens, 2009; Schoonees et al., 2017; and Goodfellow, 2004). Evidence‐informed practice educational interventions have also been used (e.g., Almost et al., 2013), although to a much smaller extent. Conducting a systematic review of currently available research offers a rigorous process for evaluating the comparative effectiveness of both evidence‐informed practice and evidence‐based practice educational interventions.

Dawes et al. (2005) and Tilson et al. (2011 have each reported on Sicily statements, which have been made about the need for developing educational interventions on evidence‐based practice in healthcare. The statements were made separately in the “Evidence‐Based Healthcare Teachers and Developers” conference held in 2003 (Dawes et al., 2005) and 2009 (Tilson et al., 2011). The statements provide suggestions for evidence‐based practice competencies, curricula, and evaluation tools for educational interventions. All health and social care students and professionals are required to understand the principles of evidence‐based practice, to have a desirable attitude toward evidence‐based practice, and to effectively implement evidence‐based practice (Dawes et al., 2005). To incorporate a culture of evidence‐based practice among health and social care students, Melnyk (2017) believes undergraduate health and social care research modules need to be based on the seven‐step model of evidence‐based practice that was developed by Melnyk et al. (2010). In addition, the curricula should include learning across the four components of evidence‐based practice, namely, knowledge, attitudes, behavior, and practice (Haggman‐Laitila et al., 2016).

Tilson et al. (2011) identified major principles for the design of evidence‐based practice evaluation tools for learners. Among the identified categories for evaluating evidence‐based practice educational interventions include the learner's knowledge of, and attitudes regarding evidence‐based practice, the learner's reaction to the educational experience, behavior congruent with evidence‐based practice as part of patient care, as well as skills in implementing evidence‐based practice. According to Tilson et al. (2011), frameworks used in assessing the effectiveness of evidence‐based practice interventions need to reflect the aims of the research module, and the aims must also correspond to the needs and characteristics of learners. For example, students may be expected to perform the seven‐steps of evidence‐based practice, whilst health practitioners may be required to acquire skills in applying evidence into practice. Tilson et al. (2011) also stated that the setting where learning, teaching, and the implementation of evidence‐based practice occur needs to be considered.

Evidence‐informed practice, on the other hand, extends beyond the initial definitions of evidence‐based practice (LoBiondo‐Wood et al., 2013) and is more inclusive than evidence‐based practice (Epstein, 2009). This is due to the following reasons. First, evidence‐informed practice recognizes practitioners as critical thinkers and encourages them to be knowledgeable about findings from all types of research (including systematic reviews, randomized controlled trials (RCTs), qualitative research, quantitative research, and mixed methods), and to utilize them in an integrative manner. Second, evidence‐informed practice considers the best available research evidence, practitioner knowledge and experience, client preferences and values, and the clinical state and circumstances (Nevo & Slonim‐Nevo, 2011). However, Melnyk and Newhouse, 2014 (p. 347) disagreed with this assertion as a difference between the two concepts. According to the authors, like evidence‐informed practice, evidence‐based practice has broadened to “integrate the best evidence for well‐designed studies and evidence‐based theories (i.e., external evidence) with a clinician's expertise, which includes internal evidence gathered from a thorough patient assessment and patient data, and a patient's preferences and values.” Although this statement may be true, the existing evidence‐based practice models (e.g., DiCenso et al., 2005; Dufault, 2004; Greenhalgh et al., 2005; Melnyk et al., 2010; Titler et al., 2001) place too much emphasis on the scientific evidence in clinical decision‐making, and give little or no attention to the other forms of evidence such as the clinical context, patient values and preferences, and practitioner's knowledge and experiences (McTavish, 2017; Miles & Loughlin, 2011).

Inasmuch as scientific evidence plays a major role in clinical decision‐making, the decision‐making process must be productive and adaptable enough to meet the on‐going changing condition and needs of the patient, as well as the knowledge and experiences of the health practitioner (LoBiondo‐Wood et al., 2013; Nevo & Slonim‐Nevo, 2011). Hence, researchers, including Nevo & Slonim‐Nevo, 2011 and McSherry, 2007, have advocated for a creative and flexible model of applying evidence into practice, where healthcare practitioners are not limited to following a series of steps (as advocated in evidence‐based practice) to apply evidence into practice. Third, unlike evidence‐informed practice, evidence‐based practice uses a formal hierarchy of research evidence, which ranks certain forms of evidence (e.g., systematic reviews and RCTs) higher than others (such as qualitative research and observational studies). Instead of the hierarchy of research evidence, proponents of evidence‐informed practice support an integrative model of practice that considers all forms of studies and prefers the evidence that provides the best answer to the clinical question (Epstein, 2009). Therefore, in place of the hierarchy of research evidence, Epstein, 2011 suggested a “wheel of evidence,” where “all forms of research, information gathering, and interpretations would be critically assessed but equally valued” (p. 225). This will ensure that all forms of evidence are considered during decision‐making in healthcare practice.

Evidence‐informed practice does not follow a stepwise approach to applying evidence into practice. According to McSherry (2007), the actual process involved in applying evidence into practice occurs in a cyclical manner, termed the evidence‐informed cycle. Similarly, Epstein (2009) described evidence‐informed practice as an integrative model that “accepts the positive contributions of evidence‐based practice, research‐based practice, practice‐based research, and reflective practice” (p. 223). Epstein (2009)'s integrative model of evidence‐informed practice is presented in the form of a Venn diagram, which highlights the commonalities and intersections among the concepts. Likewise, Moore (2016) believes evidence‐informed practice is an integration of three components, namely, evidence‐based programs, evidence‐based processes, and client and professional values. According to Moore (2016), these sources of evidence need to be blended in practice to achieve optimal person‐centered care.

Thus, an evidence‐informed practice educational intervention needs to recognize the learner as a critical thinker who is expected to consider various types of evidence in clinical decision‐making (Almost et al., 2013; McSherry et al., 2002). One is not expected to be a researcher to effectively implement evidence‐informed practice. Rather, McSherry et al. (2002) argue that the healthcare professional must be aware of the various types of evidence (such as the context of care, patient preferences, and experience, as well as the professional's own skills and expertise), not just research evidence, to deliver person‐centered care. Table 1 presents a summary of the differences and similarities between evidence‐informed practice and evidence‐based practice.

**TABLE 1:** Summary of the differences and similarities between evidence‐informed practice and evidence‐based practice

**Evidence‐based practice**	**Evidence‐informed practice**	**Similarities between evidence‐based practice and evidence‐informed practice**
Evidence‐based practice adopts a “cook‐book” approach to applying evidence into practice, and so leaves no room for flexibility (Nevo & Slonim‐Nevo, 2011).	Evidence‐informed practice recognizes practitioners as critical thinkers (McSherry, 2007; Nevo & Slonim‐Nevo, 2011), and encourages them to be creative and to consider the clinical state and circumstances when making patient care decisions.	Both evidence‐informed practice and evidence‐based practice are approaches for making informed clinical decisions (Woodbury & Kuhnke, 2014)
		Both evidence‐informed practice and evidence‐based practice integrate research with patient values and preferences and clinical knowledge and expertise (Melnyk & Newhouse, 2014)
The existing evidence‐based practice models (e.g., DiCenso et al., 2005; Dufault, 2004; Greenhalgh et al., 2005; Melnyk et al., 2010; Titler et al., 2001) rely heavily on scientific evidence, when making clinical decisions, and give little attention to other forms of evidence such as the clinical context, patient values and preferences, and practitioner's knowledge and experiences (McTavish, 2017; Miles & Loughlin, 2011)	The existing evidence‐informed practice models (e.g., McSherry, 2007; Nevo & Slonim‐Nevo, 2011) are innovative and flexible. The client is at the centre not the evidence (McTavish, 2017). One is not expected to be a researcher in order to effectively implement evidence‐informed practice; the healthcare professional must be aware of the various types of evidence, such as the context of care, patient preferences and experience, as well as the clinician's skills and expertise, not just research evidence, in order to deliver effective person‐centred care.	
Evidence‐based practice uses a formal hierarchy of research evidence, which ranks certain forms of research evidence (e.g., systematic reviews and randomized controlled trials) higher than others (such as qualitative research and observational studies).	Instead of the hierarchy of research evidence, evidence‐informed practice supports an integrative model of practice that considers all forms of research evidence (including, systematic reviews, randomized controlled trials, qualitative research, quantitative research and mixed methods), and prefers the evidence that provides the best answer to the clinical question (Epstein, 2009).	
The existing models of Evidence‐based practice adopt a stepwise approach to applying evidence into healthcare practice.	Evidence‐informed practice is an integrative (McTavish, 2017) and a systems‐based approach to applying evidence into practice, which comprises of an input, throughput and an output (McSherry, 2007)	
The linear approach of evidence‐based practice does not allow health practitioners to be creative enough, so as to meet the on‐going changing needs and conditions of the patient and the healthcare setting.	Evidence‐informed practice is adaptable, and considers the complexities of health and healthcare delivery (LoBiondo‐Wood et al., 2013; Nevo & Slonim‐Nevo, 2011). The evidence‐informed practice model considers several factors, such as the factors that influence research utilization (including workload, lack of organizational support, and time) in clinical decision‐making (McSherry, 2007).	

**Figure 1:**
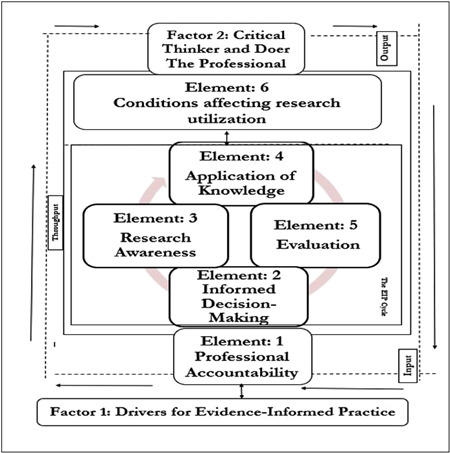
Evidence‐informed practice model

Though several models of evidence‐informed practice and evidence‐based practice exist, our operational definitions for evidence‐informed practice and evidence‐based practice educational interventions were based on McSherry (2007)'s model of evidence‐informed practice and Melnyk et al. (2010)'s model of evidence‐based practice, respectively. The following operational definitions were applied:

*Evidence‐informed practice educational interventions* referred to any formal educational program that facilitates the application of the principles of the evidence‐informed practice model developed by McSherry (2007). The evidence‐informed practice model (Figure 1), as developed by McSherry (2007) is a systems‐based model comprising input (e.g., roles and responsibilities of the health practitioner) throughput (i.e., research awareness, application of knowledge, informed decision‐making, evaluation), and output, which is an empowered professional who is a critical thinker and doer (McSherry, 2007).

*Evidence‐based practice educational interventions* referred to any formal educational program that enhances the application of the principles of the evidence‐based practice model developed by Melnyk et al. (2010). The evidence‐based practice model developed by Melnyk et al. (2010) comprises a seven‐step approach to the application of evidence into practice. These are (1) to cultivate a spirit of inquiry (2) ask a clinical question (3) search for the best evidence to answer the question (4) critically appraise the evidence (5) integrate the appraised evidence with own clinical expertise and patient preferences and values (6) evaluate the outcomes of the practice decisions or changes based on evidence and (7) disseminate evidence‐based practice results (Melnyk et al., 2010).

In this systematic review, it was not a requirement for eligible studies to mention specifically Melnyk et al. (2010)'s model of evidence‐based practice or McSherry (2007)'s model of evidence‐informed practice as the basis for the development of their educational program. However, for a study to be eligible for inclusion, it was planned that the content of its educational program(s) must include some, if not all, of the elements and/or principles of the aforementioned models.

In addition, definitions for “knowledge,” “attitudes,” “understanding,” and “behavior” were based on the Classification Rubric for Evidence‐based practice Assessment Tools in Education (CREATE) created by Tilson et al. (2011). These are provided below.

*Knowledge* referred to learners' retention of facts and concepts about evidence‐informed practice and evidence‐based practice. Hence, assessments of evidence‐informed practice and evidence‐based practice knowledge might assess a learner's ability to define evidence‐based practice and evidence‐informed practice concepts, list their basic principles, or describe levels of evidence.

*Attitudes* referred to the values ascribed by the learner to the importance and usefulness of evidence‐informed practice and evidence‐based practice to inform clinical decision‐making.

*Understanding* referred to learners' comprehension of facts and concepts about evidence‐based practice and evidence‐informed practice.

*Behavior* referred to what learners actually do in practice. It is inclusive of all the processes that a learner would use in the implementation of evidence‐informed practice and evidence‐based practice, such as assessing patient circumstances, values, preferences, and goals along with identifying the learners' own competence relative to the patient's needs to determine the focus of an answerable clinical question.

We planned that the mode of delivery of the educational program could be in the form of workshops, seminars, conferences, journal clubs, and lectures (both face‐to‐face and online). It was anticipated that the content, manner of delivery, and length of the educational program may differ in each of the studies that were to be included as there is no standard evidence‐informed practice/evidence‐based practice educational program. Evidence‐informed practice and evidence‐based practice educational interventions that are targeted toward health and social care postgraduate students or registered health and social care practitioners were excluded.

### 2.3 How the intervention might work

Most efforts to apply evidence into healthcare practice have either been unsuccessful or partially successful (Christie et al., 2012; Eccles et al., 2005; Grimshaw et al., 2004; Lechasseur et al., 2011; McTavish, 2017). The resultant effects include ineffective patient outcomes, reduced patient safety, reduced job satisfaction, and increased rate of staff turnover (Adams, 2009; Fielding & Briss, 2006; Huston, 2010; Knops et al., 2009; Melnyk & Fineout‐Overholt, 2005; Schmidt & Brown, 2007). Consequently, a lot of emphases have been placed on integrating evidence‐based practice (Masters, 2009; Melnyk, 2017; Scherer & Smith, 2002; Straus et al., 2005) and/or evidence‐informed practice competencies (Epstein, 2009; McSherry, 2007; McSherry et al., 2002; Nevo & Slonim‐Nevo, 2011) into undergraduate health and social care curricula. Yet, it remains unclear the exact components of an evidence‐based practice/evidence‐informed practice educational intervention. Healthcare educators continue to encounter challenges with regard to finding the most efficient approach to preparing health and social care students toward the application of evidence into practice (Almost et al., 2013; Flores‐ Mateo & Argimon, 2007; Oh et al., 2010; Straus et al., 2005). This has resulted in an increase in the rate and number of research investigating the effectiveness of educational interventions for enhancing knowledge, attitudes, and skills regarding, especially, evidence‐based practice (Phillips et al., 2013). There is also empirical evidence (primary studies) to support a direct link between evidence‐based practice/evidence‐informed practice educational interventions and knowledge, attitudes, understanding, and behavior, which in turn may have a positive impact on the application of evidence into practice. However, participants in most of the studies reviewed were nursing students. Some examples are given below.

Ashtorab et al. (2014) developed an evidence‐based practice educational intervention for nursing students and assessed its effectiveness, based on Rogers' diffusion of innovation model (Rogers, 2003). The authors concluded that evidence‐based practice education grounded on Roger's model leads to improved attitudes, knowledge, and adoption of evidence‐based practice. According to the authors, Rogers' diffusion of innovation model contains all the important steps that need to be applied in the teaching of evidence‐based practice.

Heye and Stevens (2009) developed an evidence‐based practice educational intervention and assessed its effectiveness on 74 undergraduate nursing students, using the Academic Center for Evidence‐based practice (ACE) Star model of knowledge transformation (Stevens, 2004). The ACE star model describes how evidence is progressively applied to healthcare practice by transforming the evidence through various stages (including translation, integration, evaluation, discovery, and summary).

Heye and Stevens (2009) indicated that the students who participated in the educational program gained research appraisal skills and knowledge in evidence‐based practice. Furthermore, the authors reported that the students acquired evidence‐based practice competencies and skills that are required for the work environment.

Several other studies have reported on the effectiveness of evidence‐based practice educational interventions and their underpinning theoretical foundations: the Self‐directed learning strategies (Fernandez et al., 2014; Kruszewski et al., 2009; Zhang et al., 2012), the Constructivist Model of learning (Fernandez et al., 2014), Bandura's self‐efficacy theory (Kim et al., 2009), as well as the Iowa model of evidence‐based practice (Kruszewski et al., 2009). Nonetheless, research in the area of evidence‐informed practice educational interventions has been limited. Almost et al. (2013) developed an educational intervention aimed at supporting nurses in the application of evidence‐informed practice. Before developing the intervention, the authors conducted interviews to examine the scope of practice, contextual setting, and learning needs of participants. A Delphi survey was then conducted to rank learning needs, which were identified by the interview participants, to select the key priorities for the intervention. The authors then conducted a pre and post‐survey, before the intervention and six months after the intervention, respectively, to assess the impact of the intervention. Thus, the development of the intervention was learner‐directed, which reaffirms McSherry (2007)'s description of the evidence‐informed practitioner as a critical thinker and doer. Unlike evidence‐based practice, practice knowledge and intervention decisions regarding evidence‐informed practice are enriched by previous research but not limited to it. In this way, evidence‐informed practice is more inclusive than evidence‐based practice (Epstein, 2009 p. 9). Nevo and Slonim‐Nevo (2011) argue that rather than focusing educational interventions on the research‐evidence dominated steps of evidence‐based practice, research findings should be included in the intervention process, but the process itself must be creative and flexible enough to meet the continually changing needs, conditions, experiences, and preferences of patients and health professionals.

**Figure 2:**
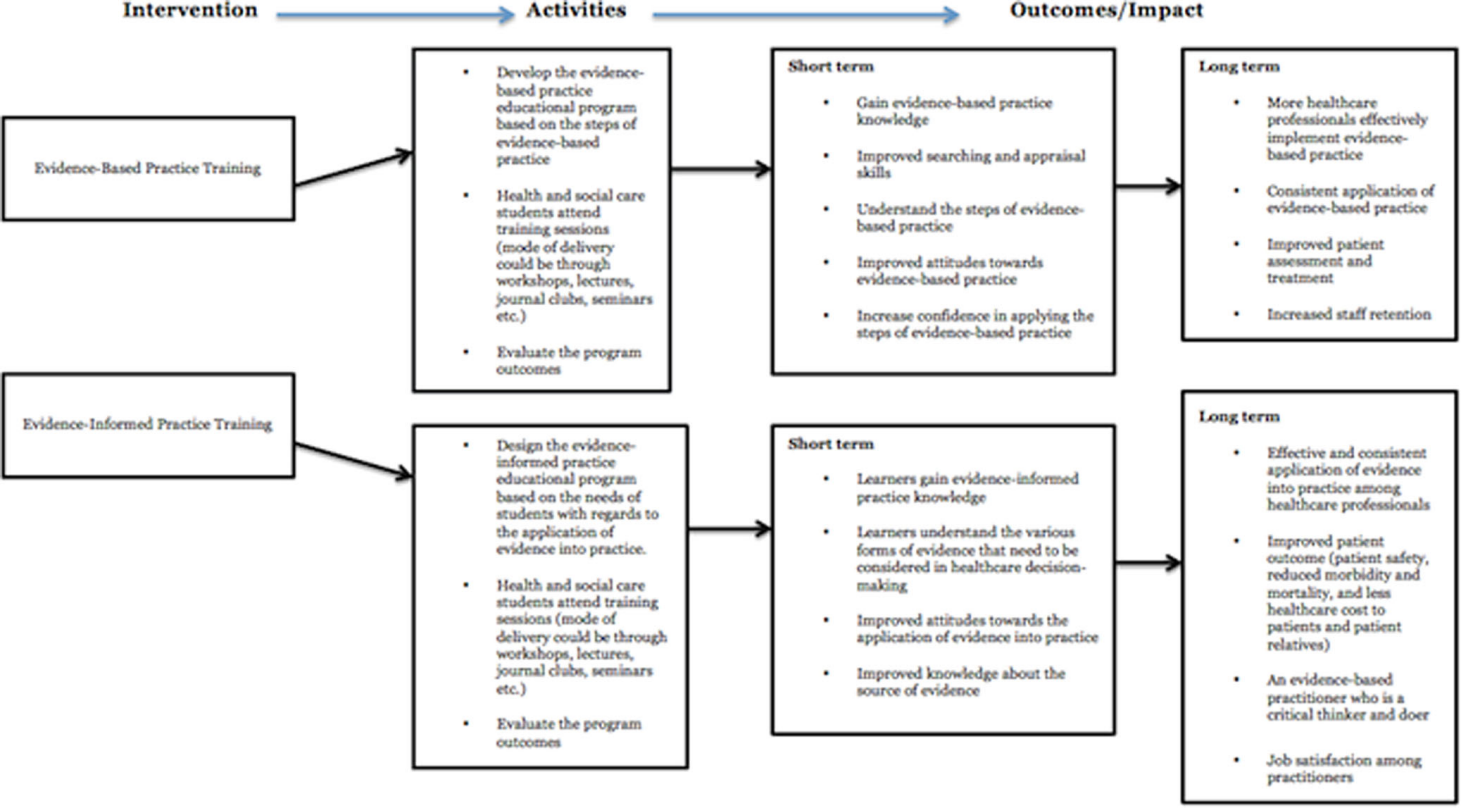
Logic model

A logic model has been presented in Figure 2 to indicate the connection between evidence‐based practice/evidence‐informed practice educational intervention and outcomes.

### 2.4 Why it is important to do this review

Despite the seeming confusion surrounding the terms “evidence‐informed practice” and “evidence‐based practice,” together with the on‐going debate in the literature as to which concept leads to better patient outcomes, no study, to the best of the researchers' knowledge, has compared through a systematic review, the effects of the two concepts on the effective implementation of evidence into practice. A review of the literature reveals several systematic reviews conducted on evidence‐based practice educational interventions and the effects of such interventions. Examples of such systematic reviews are described below.

Young et al. (2014) conducted an overview of systematic reviews that evaluated interventions for teaching evidence‐based practice to healthcare professionals (including undergraduate students, interns, residents, and practicing healthcare professionals). Comparison interventions in the study were no intervention or different strategies. The authors included 15 published and 1 unpublished systematic reviews. The outcome criteria included evidence‐based practice knowledge, critical appraisal skills, attitudes, practices, and health outcomes. In many of the included studies, however, the focus was on critical appraisal skills. The systematic reviews that were reviewed used a number of different educational interventions of varying formats (e.g., lectures, online teaching, and journal clubs), content, and duration to teach the various component of evidence‐based practice in a range of settings. The results of the study indicated that multifaceted, clinically integrated interventions (e.g., lectures, online teaching, and journal clubs), with assessment, led to improved attitudes, knowledge, and skills toward evidence‐based practice. The majority of the included systematic reviews reported poorly the findings from the source studies, without reference to significant tests or effect sizes. Moreover, the outcome criteria (e.g., knowledge, skills, attitudes, practices, and health outcomes) were described narratively as improved or not, with the use of vote counting.

Coomarasamy and Khan (2004) conducted a systematic review to evaluate the effects of standalone versus clinically integrated teaching in evidence‐based medicine on postgraduate healthcare students' knowledge, critical appraisal skills, attitudes, and behavior. The results indicated that standalone teaching improved knowledge, but not skills, attitudes, or behavior. Clinically integrated teaching, however, improved knowledge, skills, attitudes, and behavior. A similar systematic review by Flores‐ Mateo and Argimon (2007) identified a small significant improvement in postgraduate healthcare students' skills, knowledge, behavior, and attitudes after participating in evidence‐based practice interventions. Furthermore, a systematic review of the literature has been conducted to identify the effectiveness of evidence‐based practice training programs and their components for allied health professionals (Dizon et al., 2012). The researchers reported that irrespective of the allied health discipline, there was consistent evidence of significant changes in knowledge and skills among health practitioners, after participating in an evidence‐based practice educational program. In addition, recently, a systematic review has been conducted by Rohwer et al. (2017) to assess the effectiveness of e‐learning of evidence‐based practice on increasing evidence‐based practice competencies among healthcare professionals (medical doctors, nurses, physiotherapists, physician assistants, and athletic trainers). The results showed that pure e‐learning compared to no learning led to an improvement in knowledge as well as attitudes regarding evidence‐based practice among the various professional groups.

Yet, according to a comprehensive literature review, no specific systematic review has been conducted on evidence‐informed practice educational interventions and the effects of such interventions on the knowledge, attitudes, understanding, and behavior of undergraduate health and social care students. Two reviews (conducted by McCormack et al., 2013, and Yost et al., 2015) on evidence‐informed practice interventions were identified in the literature. However, these reviews focused on “change agency” and “knowledge translation” as interventions in improving evidence‐informed practice. For example, McCormack et al. (2013) conducted a realist review of strategies and interventions to promote evidence‐informed practice, but the authors focused only on “change agency” as an intervention aimed at improving the efficiency of the application of evidence. Also, a systematic review by Yost et al. (2015) concentrated on the effectiveness of knowledge translation on evidence‐informed decision‐making among nurses. Moreover, a relatively recent systematic review by Sarkies et al. (2017) focused on evaluating the effectiveness of research implementation strategies for promoting evidence‐informed policy and management decisions in healthcare. The authors also described factors that are perceived to be associated with effective strategies and the correlation between these factors. Nineteen papers (research articles) were included in Sarkies et al. (2017)'s review. The results revealed a number of implementation strategies that can be used in promoting evidence‐informed policy and management in healthcare. The strategies included workshops, knowledge brokering, policy briefs, fellowship programs, consortia, literature reviews/rapid reviews, multi‐stakeholder policy dialogue, and multifaceted strategies. It is important to note that these strategies, though relevant, are more linked to healthcare management and policy decisions rather than typical patient care decision‐making/healthcare practice, which is the focus of the present systematic review.

This systematic review offers originality and is significantly different from previously conducted systematic reviews in three ways. First, this review focused on pre‐registration undergraduate health and social care students as opposed to only nursing students, nurses, or health care professionals. Second, the current review assessed the effectiveness of evidence‐informed practice educational interventions, while recent studies by Rohwer et al. (2017) and Yost et al. (2015) assessed the effectiveness of e‐learning of evidence‐based health care and the effectiveness of knowledge translation on evidence‐informed decision‐making, respectively. Third, this systematic review focused on comparing the effectiveness of evidence‐informed practice to evidence‐based practice educational interventions on undergraduate pre‐registered health and social care students' knowledge, attitudes, understanding, and behavior regarding the application of evidence into practice. The current review also aimed to determine whether evidence‐informed practice and evidence‐based practice act together, or individually to facilitate the application of evidence into practice.

By conducting a comprehensive systematic review of the literature that specifically compares the effectiveness of evidence‐informed practice to evidence‐based practice educational interventions on undergraduate health and social care students, we hoped to review and analyze current evidence‐informed practice and evidence‐based practice approaches in higher education settings. In addition, we hoped that the results of this systematic review would help to determine the relative effectiveness of evidence‐informed practice and evidence‐based practice educational interventions, as well as identify gaps in the current literature. We hoped to be able to offer direction for practice, policy, and future inquiry in this growing area of research and practice.

## 3 OBJECTIVES

The primary objective of this systematic review is as follows.

- To evaluate and synthesize literature on the relative effectiveness of evidence‐informed practice and evidence‐based practice educational interventions for improving knowledge, attitudes, understanding, and behavior of undergraduate pre‐registration health and social care students regarding the application of evidence into practice.

Specifically, the review aimed to address the following research questions:

1. Is there a difference (i.e., difference in content, outcome) between evidence‐informed practice and evidence‐based practice educational interventions?
2. Does participating in evidence‐informed practice educational interventions relative to evidence‐based practice educational interventions facilitate the application of evidence into practice (as measured by, for example, self‐reports on effective application of evidence into practice)?
3. Do both evidence‐informed practice and evidence‐based practice educational interventions targeted at undergraduate health and social care students influence patient outcomes (as measured by, e.g., reduced morbidity and mortality, absence of nosocomial infections)?
4. What factors affect the impact of evidence‐informed practice and evidence‐based practice educational interventions (as measured by, e.g., course content, mode of delivery, multifaceted interventions, standalone intervention)?

## 4 METHODS

### 4.1 Criteria for considering studies for this review

#### 4.1.1 Types of studies

This review followed standard procedures for conducting and reporting systematic literature reviews. The protocol for this systematic review (Kumah et al., 2019) was published in July 2019. The protocol is available at: https://doi.org/10.1002/cl2.1015.

In this review, we intended to include both qualitative and quantitative primary studies (a mixed‐methods systematic review) that compared evidence‐informed practice educational interventions with evidence‐based practice educational interventions.

We planned to include quantitative primary studies that used both experimental and epidemiological research designs such as RCTs, non‐RCTs, quasi‐experimental, before and after studies, prospective and retrospective cohort studies.

We also planned to include qualitative primary studies that had used descriptive epidemiological study designs. Examples include case series, individual case reports, descriptive cross‐sectional studies, focus groups, and interviews. Furthermore, we intended to include primary studies that have used qualitative approaches such as ethnography, phenomenology, and grounded theory. We planned to discuss the biases and limitations associated with any included study design in relation to the impact it may have on the effectiveness of the intervention.

**Figure 3:**
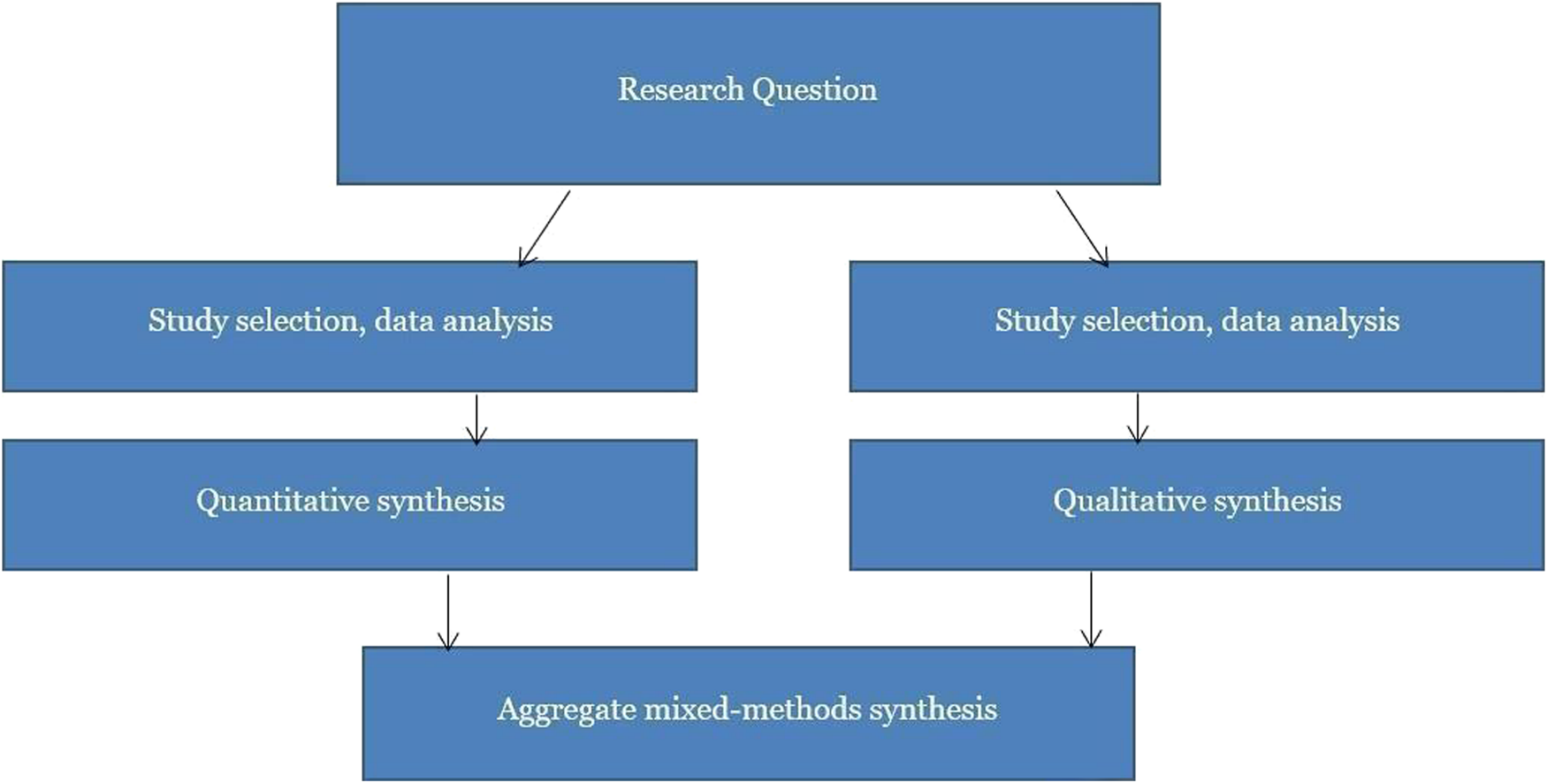
Summary of mixed‐methods strategy to be employed

For the primary analysis, the intention was to follow the recommended steps by Sandelowski et al. (2012): to first conduct two separate syntheses for included quantitative and qualitative primary studies. We planned to synthesize qualitative studies by way of meta‐aggregation and quantitative studies by way of meta‐analysis (Lockwood et al., 2015). We planned to then integrate the results of the two separate syntheses by means of an aggregative mixed‐methods synthesis. We intended to integrate the two results (i.e., qualitative and quantitative results) by translating findings from the quantitative synthesis into qualitative statements, by the use of Bayesian conversion (Joanna Briggs Institute, 2014). Figure 3 presents the mixed‐methods approach we intended to employ in this systematic review.

#### 4.1.2 Types of participants

We intended to include undergraduate pre‐registration health and social care students in higher education (University) from any geographical area. We planned to include undergraduate pre‐registration students studying health and social care programs such as nursing, midwifery, dental hygiene, and dental therapy, dental nurse practice, diagnostic radiography, occupational therapy, operating department practice studies, paramedic practice, social work, and physiotherapy.

We planned to exclude studies whose participants were registered health and social care practitioners and postgraduate students.

#### 4.1.3 Types of interventions

The intention was to include primary studies that evaluate and compare any formal evidence‐based practice educational intervention with evidence‐informed practice educational interventions aimed at improving undergraduate pre‐registration health and social care students' knowledge, attitudes, understanding, and behavior regarding the application of evidence into healthcare practice. Such interventions may be delivered via either workshops, seminars, conferences, journal clubs, or lectures (both face‐to‐face and online).

Specifically, we planned to include any formal educational intervention that incorporates any or all of the principles and elements of McSherry (2007)'s evidence‐informed practice model and Melnyk et al., 2010's evidence‐based practice model. It was not a requirement for eligible studies to mention specifically Melnyk et al. (2010)'s model of evidence‐based practice or McSherry (2007)'s model of evidence‐informed practice as the basis for the development of their educational program. However, we planned that for a study to be eligible, the content of its educational program must include some, if not all, of the elements and/or principles of the models. We anticipated that the content, manner of delivery, and length of the educational program may differ in eligible studies as there is no standard evidence‐informed practice/evidence‐based practice educational program.

Evidence‐informed practice and evidence‐based practice educational interventions that are targeted at health and social care postgraduate students or registered health and social care practitioners were excluded. We intended to include, as comparison conditions, educational interventions that do not advance the teaching of the principles and processes of evidence‐informed practice, and/or evidence‐based practice in healthcare.

#### 4.1.4 Types of outcome measures

Outlined below are the primary and secondary outcome measures for this systematic review

##### Primary outcomes

1. Participants' knowledge about evidence‐informed practice and/or evidence‐based practice.
2. Participants' understanding of evidence‐informed practice and/or evidence‐based practice.
3. Participants' attitudes toward evidence‐informed practice and/or evidence‐based practice.
4. Participants' behavior toward evidence‐informed practice and evidence‐based practice.

Since there is no uniform tool for evaluating the effectiveness of evidence‐based practice and evidence‐informed practice educational interventions, we planned that measurement of the above outcomes may be conducted using standardized or unstandardized instruments. Some examples of these instruments include:

- the use of a standardized questionnaire to evaluate knowledge, attitude, understanding, and behavior toward the application of evidence into practice. Examples of such questionnaires include (1) the Evidence‐Based Practice Belief (EBPB) and Evidence‐Based Practice Implementation (EBPI) scales developed by Melnyk et al. (2008). The EBPB scale is a 16‐item questionnaire that allows measurement of an individual's belief about the values of evidence‐based practice and the ability to implement evidence‐based practice, whereas the EBPI scale is an 18‐item questionnaire that evaluates the extent to which evidence‐based practice is implemented, (2) the use of pre and post validated instruments such as the Berlin test (Fritsche et al., 2002) to measure changes in knowledge, and (3) the use of Likert scale questions to measure changes in attitudes before and after the intervention.
- unstandardized instruments include self‐reports from study participants and researcher‐administered measures.

##### Secondary outcomes

The intention was to include studies that measure the impact of evidence‐informed practice and/or evidence‐based practice educational programs on patient outcomes. We planned to assess patient outcome indicators such as user experience, length of hospital stays, absence of nosocomial infections, patient and health practitioner satisfaction, mortality, and morbidity rates.

##### Duration of follow‐up

No limit was placed on the duration of follow‐up. The rationale was to give room for studies with either short‐ or long‐term follow‐up duration to be eligible for inclusion.

##### Types of settings

We intended to include primary studies from any geographical area. However, due to language translation issues, we planned to include only studies written in English. We also planned that studies whose title and abstracts are in English and meet the inclusion criteria, but the full article is reported in another language would be included, subject to the availability of translation services.

##### Time

To qualify for inclusion in this systematic review, studies must have been published during the period from 1996 (the date when evidence‐based practice first emerged in the literature) (Closs & Cheater, 1999; Sackett et al., 1996), to the date when the literature search was concluded (June 17, 2019).

### 4.2 Search methods for identification of studies

#### 4.2.1 Search terms and keywords

We used a combination of keywords and terms related to the population, intervention, outcome, and study design to conduct the search. Specific strategies for each database were explored, such as the use of Boolean operators (e.g., OR, AND), wildcards (such as?), phrase operators (e.g. “”), and truncations (including *). This was done to ensure search precision and sensitivity. In addition, we used three sets of terms for the search strategy: the population, intervention(s), and outcomes. We used limiting commands to narrow the results by date and type of study design. The search was limited to studies published from 1996, which is the year when evidence‐based practice first emerged in the literature. No limit was placed on the language of publication, however, due to language translation issues, eligible studies whose full texts were not in English might have been included only if there were available language translation facilities.

Below are examples of the search terms used in the current review:

- *Targeted population*: nurs* OR physio* OR “occupa* therap*” OR “dental Hygiene” OR “undergraduate healthcare student*” OR “undergraduate social care student*” OR baccalaureat* OR “social work” OR dent* OR BSc OR student* OR “higher education” OR “undergrad* nurs* student*”
- *Intervention*: evidence‐informed* OR evidence‐based* OR “evidence‐informed practice” OR “evidence‐based practice” OR EBP OR EIP OR “evidence‐informed practice education” OR “evidence‐based practice education” OR “evidence into practice” OR evidence‐informed near. practice teaching learning OR evidence‐based near. practice teaching learning
- *Outcomes*: “knowledge, attitudes, understanding and behavio* regarding EBP” OR “knowledge near. attitudes understanding behavio* regarding EIP OR “Knowledge of evidence‐informed*” OR “knowledge of evidence‐based*” OR “patient outcome*” OR outcome*
- *Study design/type*: trial* OR “randomi?ed control trial” OR “qua?i‐experiment*” OR random OR experiment OR “control* group*” OR program OR intervention OR evaluat* OR qualitative OR quantitative OR ethnograpy OR “control* study” OR “control* studies” OR “control* design*” OR “control* trial*” OR “control group design” OR RCT OR rct OR “trial registration”

#### 4.2.2 Management of references

We exported the full set of search results directly into an Endnote X9 Library. Where this was not possible, search results were manually entered into the Endnote Library. The Endnote library made it easier to identify duplicates and manage references.

#### 4.2.3 Search strategy

The search to identify eligible studies was initially carried out in June 2018, and then a repeat search was conducted in June 2019. We utilized a number of strategies, to identify published and unpublished studies that meet the inclusion criteria described above. These strategies are outlined below.

##### Electronic searches

The following electronic searches were conducted to identify eligible studies.

1. An electronic database search was conducted using the following databases.

- Academic Search complete
- Academic search premier
- AMED
- Australian education index
- British education index
- Campbell systematic reviews
- Canada bibliographic database (CBCA Education)
- CINAHL
- Cochrane Library
- Database of Abstracts of Reviews on Effectiveness
- Dissertation Abstracts International
- Education Abstracts
- Education complete
- Education full text: Wilson
- ERIC
- Evidence‐based program database
- JBI database of systematic reviews
- Medline
- PsycInfo
- Pubmed
- SciELO (Scientific Electronic Library Online)
- Scopus

Supporting Information Appendix 1 presents the search strategy for the MEDLINE database searched on the EBSCOhost platform. We modified the search terms and strategies for the different databases.

2. A web search was conducted using the following search engines.

- Google
- Google Scholar

3. A gray literature search was conducted using the following databases.

- OpenGrey (System for Information on Grey Literature in Europe)
- System for information on Grey Literature
- The Society for Research on Educational Effectiveness
- Virginia Henderson Global Nursing e‐Repository

##### Searching other resources

The following strategies were also used to identify eligible studies.

- Hand searching: the table of contents of three journals were hand‐searched for relevant studies. The journals include the *Worldviews on Evidence‐Based Nursing Journal*, the *British Medical Journal*, and the *British Journal of Social Work*.
- Tracking bibliographies of previously retrieved studies and literature reviews: we screened the reference list of previously conducted systematic reviews, meta‐analysis, and primary studies for relevant studies.

### 4.3 Data collection and analysis

#### 4.3.1 Selection of studies

First, we exported search results from the various databases and search engines into an Endnote X9 software. Second, we searched for and removed duplicates using the Endnote software. Third, we exported search results from Endnote into Covidence (a web‐based software platform that streamlines the production of systematic reviews) for the screening of the search results. Two authors independently screened the titles and abstracts for relevant papers. The full text of potentially relevant papers was subsequently assessed by two independent authors for inclusion. Disagreements were resolved through discussion. Where disagreements persisted, a third reviewer was contacted. We selected papers that were published during the period from 1996 (the date when evidence‐based practice first emerged in the literature) (Closs & Cheater, 1999; Sackett et al., 1996) to the date when the literature search was concluded (June 2019). A total of 46 full‐text papers were screened for eligibility. Among the 46 papers, 45 papers had assessed solely evidence‐based practice educational interventions and 1 paper had assessed the effectiveness of evidence‐informed practice interventions. However, we identified no evidence of primary studies that had evaluated and compared the effectiveness of evidence‐informed practice to evidence‐based practice educational interventions. As such, we were unable to perform most of the pre‐stated methodology. We will, therefore, describe the planned methodologies in the ensuing sections.

#### 4.3.2 Data extraction and management

Had we found any eligible study, two independent authors (either S. H. and R. M. or E. A. K. and J. B. S.) would have assessed its methodological validity using standardized critical appraisal instruments from the Joanna Briggs Institute Meta‐Analysis of Statistics Assessment and Review Instrument (JBI‐MAStARI). We would have used the JBI‐MAStARI checklist for case‐control studies, the checklist for case reports, the checklist for cohort studies, the checklist for quasi‐experimental, the checklist for RCTs, and the checklist for analytical cross‐sectional studies. Disagreements between authors would have been resolved through discussion; if no agreement could be reached, a third author was to be consulted.

Data would have been extracted from included papers using standardized data extraction form for intervention reviews for RCTs and non‐RCTs developed by the Cochrane Collaboration. We would have extracted information relating to study design, interventions, population, outcomes that are of significance to the review questions, and specific objectives, and methods used to assess the impact of evidence‐informed practice/evidence‐based practice educational interventions on patient outcomes. Supporting Information Appendix 2 presents the data extraction form we planned to use for this review.

#### 4.3.3 Assessment of risk of bias in included studies

Two authors (either R. M. and S. H. or E. A. K. and J. B. S.) would have independently assessed eligible studies for risk of bias. This would have been done using the Cochrane Collaboration's Risk of Bias tool (Higgins & Green, 2011). Discrepancies between reviewers would have been resolved through consultation and discussion with a third author (V. W.). We planned to categorize studies as having high, low, or unclear risks of bias. We planned to use the following criteria to assess the risk of bias:

##### Random sequence generation

We would have categorized studies as having a high risk of bias if the authors used a nonrandom sequence generation process, for example, the sequence generated by the preference of the study participants, even or odd date of birth, or availability of the intervention. Studies would have been judged as having a low risk of bias if a random sequence generation process was used, and the process used in generating the allocation sequence is described in sufficient detail and able to produce comparable groups.

##### Allocation concealment

Studies would have been deemed as having a low risk of bias if the method used in generating the allocation sequence was adequately concealed from study participants, such that study participants are unable to foresee group allocation. We planned to judge studies as having a high risk of bias if the process used in generating allocation sequence was open such that study participants can predict group allocation. This introduces selection bias. An example includes using a list of random numbers.

##### Blinding of participants and personnel

Inadequate blinding results in participants and personnel having different expectations for their performance, hence biasing the results of the trial. We planned to consider studies as having a low risk of bias if participants and trial personnel are blind to allocation status.

##### Blinding of outcomes assessors

We planned to examine included studies to determine if outcome assessors were blind to allocation status. Studies would have been considered as having a low risk of bias if outcomes are assessed by independent investigators who had no previous knowledge of group allocation.

##### Incomplete outcome data

We planned to assess studies to determine if there are any missing outcome data. We would have examined the differences between intervention and control groups in relation to measurement attrition and the reasons for missing data. Studies with low attrition (<20%), no attrition, or no evidence of differential attrition would have been considered as having a low risk of bias. We planned to record the use of Intention to Treat (ITT) analysis and methods of account for missing data (e.g., using missing multiple imputations).

##### Selective outcome reporting

We intended to assess studies for reporting bias to determine whether there are inconsistencies between measured outcomes and reported outcomes. Studies would have been considered as having a low risk of bias if the results section of publications clearly show that all pre‐specified outcomes are reported.

#### 4.3.4 Measures of treatment effect

##### Continuous data

For continuous data, where outcomes on the same scale are presented, we planned to use mean difference, with a 95% confidence interval. However, where outcome measures differ between studies, we would have used the standardized mean difference as the effect size metric based on Hedges' *g*, which is calculated using the following formula:

SMD = Difference in mean outcome between groups/Standard deviation of outcome among participants.

##### Dichotomous data

For dichotomous data, we planned to calculate the risk ratio (and its 95% confidence interval) for the occurrence of an event. For meta‐analysis, we planned to convert risk ratios to the standardized mean difference, using David Wilson's practical effect size calculator. We intended to use meta‐regression to assess the impact of moderator variables on the effect size of interventions. We planned to conduct moderator analysis if a reasonable number of eligible research articles were identified and if the required data is presented in the report.

##### Studies with multiple groups

For studies with one control group versus two or more intervention groups, and all the interventions are regarded as relevant to the study, we planned to use the following options: (1) if the intervention groups are not similar, we would have divided the sample size of the control group into two (or more based on the number of intervention groups), and then compared with the intervention groups (2) if the intervention groups were similar, we would have treated the two groups as a single group. Therefore, we would have provided two effect size estimates in this study. This was to ensure that participants in the control group were not “double‐counted” (Higgins & Green, 2011). We planned to employ a similar approach, but in reverse, in the event that an included study has one intervention group but two control groups. We also planned that if an included study contained an irrelevant and relevant intervention group, we would have included only data from the relevant intervention group for analysis.

#### 4.3.5 Unit of analysis issues

In this systematic review, it was anticipated that included studies may have either involved individual participants or clusters (groups) of participants as units of analysis. we planned that in the event that cluster‐randomized trials (i.e., studies where participants are allocated as a group rather than as individuals) are identified as eligible, we would have used standard conversion criteria as recommended in the Cochrane Handbook (Higgins & Green, 2011). We planned to do this only if such studies have not been properly adjusted for clustering (e.g., by the use of multi‐level modeling or robust standard errors).

The Cochrane Handbook (Higgins & Green, 2011) recommends guidelines to be followed in calculating the effective sample size in a cluster‐randomized trial. According to the Handbook, the effective sample size can be calculated by dividing the original sample size by the design effect. This equals 1 + (*M* − 1) **×** ICC, where *M* is the average cluster size and ICC is the Intra‐cluster Correlation Coefficient.

#### 4.3.6 Dealing with missing data

We planned to contact the first author of studies with incomplete reports on data or to request relevant information that is missing from the report.

We planned that if requested data was not provided, our options for dealing with missing data would be based on whether data is “missing at random” or “missing not at random.” We planned that if data were missing at random (i.e., if the fact that they are missing is unrelated to actual values of the missing data), data analysis would have been conducted based on the available data.

However, if data is missing not at random (i.e., if the fact that they are missing is related to the actual missing data), we planned to impute the missing data with replacement values, and treat these values as if they were observed (e.g., last observation carried forward, imputing an assumed outcome such as assuming all were poor outcomes, imputing the mean, imputing based on predicted values from a regression analysis).

#### 4.3.7 Assessment of heterogeneity

We intended to assess heterogeneity through the comparison of factors such as participant demographics, type of intervention, type of control comparators, and outcome measures. We would have assessed and reported heterogeneity visually and by examining the *I* ^2^ statistic, which describes the approximate proportion of variation that is due to heterogeneity rather than sampling error. This would have been supplemented by the *χ* ^2^ test, where a *p* value < 0.05 indicates heterogeneity of intervention effects. In addition, we planned to estimate and present *τ* ^2^ along with its CIs, as an estimate of the magnitude of variation between studies. This would have provided an estimate of the amount of between‐study variation. We also planned to use sensitivity and subgroup analyses to investigate possible sources of heterogeneity.

#### 4.3.8 Assessment of reporting biases

We planned to assess studies for reporting bias to determine whether there were inconsistencies between measured outcomes and reported outcomes. Studies would have been considered as having a low risk of bias if the results section of publications clearly showed that all pre‐specified outcomes are reported.

#### 4.3.9 Data synthesis

We planned to use narrative and statistical methods to synthesize included studies. The synthesis would have focused on calculating the effect sizes of the included studies. We planned to conduct meta‐analysis if our search yielded sufficient (i.e., two or more) eligible studies that can be grouped together satisfactorily. A logical approach would have been used when combining studies in meta‐analysis. Had we found any eligible studies, decisions on combining studies in meta‐analysis would have been based on two reasons: (1) a sufficient number of eligible studies with similar characteristics, (2) similar characteristics shared by those eligible studies may include the type of intervention and the targeted outcome of the intervention. Had we conducted a meta‐analysis, we planned to use the comprehensive meta‐analysis software developed by Borentein et al. (2005). We would have conducted separate analyses for primary outcomes (i.e., knowledge, attitudes, behavior, and understanding) and secondary outcomes (i.e., patient outcome). In addition, separate analyses would have been conducted for the effect of evidence‐based practice and evidence‐informed practice interventions. We planned to compare evidence‐based practice and evidence‐informed practice interventions by conducting a mean comparison test between the two concepts. The intervention versus control comparisons for each of the concepts would have been based on adjusted post‐test means that control for imbalance at pre‐test. If this information was not available, we planned to subtract the pre‐test means effect size from the post‐test mean effect size by using the unadjusted pooled standard deviation.

#### 4.3.10 Subgroup analysis and investigation of heterogeneity

Where there was significant statistical heterogeneity, we planned to conduct subgroup analysis to consider the effects of variables, such as participant's age, geographical area, mode of delivery and content of the educational intervention, and type of study design.

#### 4.3.11 Sensitivity analysis

Had we found any eligible studies, we planned to conduct sensitivity analysis to determine whether the overall results of data analysis was influenced by the removal of:

- Unpublished studies
- Studies with outlier effect sizes
- Studies with high risks of bias
- Studies with missing information (e.g., incomplete presentation of findings)

#### 4.3.12 Treatment of qualitative research

##### Assessment of methodological quality of qualitative papers

Two authors (either S. H. and R. M. or E. A. K. and J. B. S.) would have independently assessed included qualitative studies for methodological validity using the JBI Qualitative Assessment and Review Instrument (JBI‐QARI). Any disagreements between authors would have been resolved through discussion, if no agreement could be reached, a third author would have been consulted.

##### Data extraction and management

We planned to extract data from included papers using the Cochrane Collaboration's Data Collection form for Intervention Reviews for RCTs and non‐RCTs (Supporting Information Appendix 2). We would have extracted data relating to the population, study methods, details about the phenomena of interest, outcomes of significance to the review question and specific objectives, and methods used to assess the impact of evidence‐informed practice/evidence‐based practice educational interventions on patient outcomes.

##### Data synthesis and analysis

We planned to pool qualitative research findings using JBI‐QARI. This would have involved the synthesis or aggregation of findings to generate a set of statements that represent the aggregation. Findings would have been assembled based on their quality, and, by grouping findings with similar meanings together. We would have then performed a meta‐synthesis of these groups or categories to produce a single set of comprehensive synthesized findings. If textual pooling was not possible, we would have presented findings in narrative form.

Finally, we planned to integrate results from both the quantitative review and the qualitative review using the JBI Mixed Methods Aggregation Instrument (MMARI). We intended to achieve integration by translating findings from the quantitative review into qualitative results using Bayesian conversion to generate synthesized results.

#### 4.3.13 Summary of findings and assessment of the certainty of the evidence

Not applicable to the current review.

## 5 RESULTS

### 5.1 Description of studies

The findings of the current systematic review are presented below.

#### 5.1.1 Results of the search

**Figure 4:**
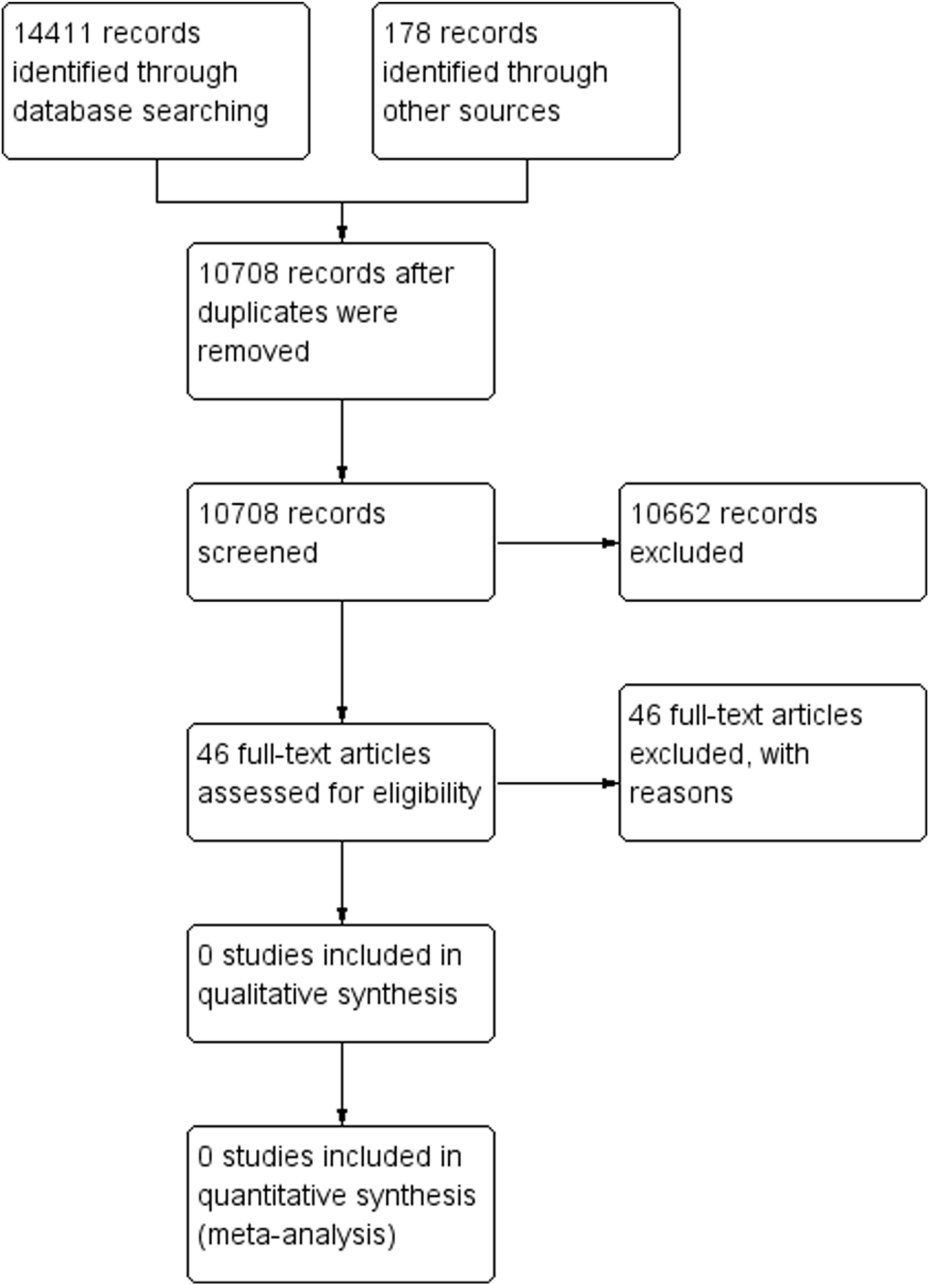
Study flow diagram

Database searches were conducted by a librarian (JH) and another review author (EK). Terms and keywords specific to the Participant (P), Intervention (I), Comparative intervention (C), and the Outcome (O) were used to generate relevant articles. A total of 14,411 citations were identified through a database search (completed on 17 June 2019). A search of additional records including google and google scholar yielded 178 citations. After duplicates were removed, 10708 records were retained for screening. After the screening, a total of 46 full‐text studies were assessed for eligibility. Among the 46 studies, 45 had assessed solely evidence‐based practice educational interventions and 1 paper had assessed the effectiveness of evidence‐informed practice interventions. However, we found no evidence of primary studies that had evaluated and compared the effectiveness of evidence‐informed practice educational interventions with evidence‐based practice educational interventions. A summary of the search results is presented in Figure 4 (flow diagram).

#### 5.1.2 Included studies

We did not identify any qualitative nor quantitative study that was eligible for inclusion in this review.

#### 5.1.3 Excluded studies

We evaluated and excluded 46 full‐text articles. The rationale for exclusion was that none of the 46 studies had evaluated and compared the effectiveness of evidence‐informed practice educational interventions with evidence‐based practice educational interventions. Out of the 46 articles, 45 had evaluated solely, the effectiveness of evidence‐based practice educational interventions and 1 article was on evidence‐informed practice educational intervention. Hence, these articles were excluded as they did not meet the inclusion criteria. Refer to the Summary of findings table for further details regarding the characteristics of excluded studies.

### 5.2 Risk of bias in included studies

We evaluated no study for methodological quality.

#### 5.2.1 Allocation (selection bias)

We found no study eligible for inclusion in this review.

#### 5.2.2 Blinding (performance bias and detection bias)

We found no study eligible for inclusion in this review.

#### 5.2.3 Incomplete outcome data (attrition bias)

We found no study eligible for inclusion in this review.

#### 5.2.4 Selective reporting (reporting bias)

We found no study eligible for inclusion in this review.

#### 5.2.5 Other potential sources of bias

We found no study eligible for inclusion in this review.

### 5.3 Effects of interventions

We found no study eligible for inclusion in this review.

## 6 DISCUSSION

### 6.1 Summary of main results

We did not identify any evidence on the relative effectiveness of evidence‐informed practice and evidence‐based practice educational interventions for improving knowledge, attitudes, understanding, and behavior of undergraduate pre‐registration health and social care students toward the application of evidence into practice.

### 6.2 Overall completeness and applicability of evidence

This review is considered an empty review, as we found no study that met all the inclusion criteria. This notwithstanding, the review offers vital evidence concerning the gaps in the literature on the effectiveness of evidence‐informed practice versus evidence‐based practice educational interventions, and whether the two concepts act together or individually to facilitate the application of evidence into healthcare practice. There are a plethora of studies that have evaluated the effectiveness of evidence‐based practice educational interventions on undergraduate pre‐registration health and social care students (this review identified 45 primary studies), however, there is limited evidence on the effectiveness of evidence‐informed practice educational interventions as well as a comparison of the effectiveness of the two concepts.

Evidence‐informed practice is evolving, as understanding and expertise increase (WHO, 2017). In recent times, there has been lots of attention on evidence‐informed practice, particularly due to the challenges associated with the implementation of evidence‐based practice, which has created a gap between evidence and healthcare practice. To bridge this gap, it is imperative to invest in other alternatives to applying evidence into practice. Most researchers (examples include Epstein, 2009; Epstein, 2011; Nevo & Slonim‐Nevo, 2011) have consistently called for a change of term from “evidence‐based practice” to “evidence‐informed practice,” arguing that practice founded on the concept of evidence‐informed practice results in better patient outcomes. However, it is not just enough to call for a change of terms without relevant empirical evidence demonstrating the effectiveness of evidence‐informed practice compared to evidence‐based practice in facilitating the application of evidence into practice. Although this evidence is not yet available, it is within the reach of current methodologies. No primary study had evaluated the effects of evidence‐informed practice educational interventions on undergraduate health and social care students' knowledge, attitudes, understanding, and behavior. Nonetheless, the majority of the full‐text primary studies that were assessed in this review measured, as outcomes, the effects of evidence‐based practice interventions on either knowledge, attitudes, understanding, or behavior of undergraduate health and social care students. Some examples of such studies include Boruff and Thomas (2011), Cosme et al. (2018), Jalali‐Nia et al. (2011), Lewis et al. (2016), Long et al. (2011), McEvoy et al. (2018), Orta et al. (2016), Ruzafa‐Martinez et al. (2016), Santiago et al. (2018), Scurlock‐Evans et al. (2017), Serfass and Hagedorn Wonder (2018), and Kim et al. (2009).

To prepare health and social care students to effectively apply evidence into practice, it is imperative that undergraduate health and social care curricula are designed to enhance progressive knowledge development on the application of evidence into clinical practice. Moreover, students' experiences in the clinical setting must produce opportunities for them to use evidence in patient care decisions as well as at the organizational level to impact patient outcomes.

The findings of this systematic review reveal the need for rigorously designed studies that are informed by empirical and theoretical literature on evidence‐informed practice versus evidence‐based practice educational interventions. The characterizations of the processes involved in implementing evidence‐informed practice and evidence‐based practice given in this systematic review offer intervention designs based on existing empirical and theoretical literature, which could be considered in the design of evidence‐informed practice and evidence‐based practice educational interventions.

### 6.3 Quality of the evidence

We searched the literature for both quantitative and qualitative studies on the effectiveness of evidence‐informed practice versus evidence‐based practice educational interventions on the knowledge, attitudes, understanding, and behavior of undergraduate pre‐registration health and social care students. Although there is some evidence of studies that have assessed the effectiveness of either evidence‐based practice or evidence‐informed practice educational interventions, our search revealed no evidence of studies that compared the effectiveness of evidence‐informed practice with evidence‐based practice educational interventions. Thus, we are unable to draw any conclusions regarding the effectiveness of these two concepts when compared to each other. The evidence is current to June 17, 2019.

### 6.4 Potential biases in the review process

We made every effort to minimize bias in this review. A comprehensive search of multiple health and social care databases was conducted, and no limit was applied to publication status or language. The search strategy was developed by an experienced health and social care librarian, in consultation with experts of the review team. The search for papers was done by two review authors (a librarian, J. H., and E. A. K.). Screening of the search results was also conducted by two independent review authors (E. A. K. and J. B. S.). Disagreements were resolved by another review author (R. M.).

The main potential bias of this review is the unlikely event that we have missed any study that evaluated and compared the effectiveness of evidence‐informed practice to evidence‐based practice educational interventions on the knowledge, attitudes, understanding, and behavior of undergraduate health and social care students. However, given our extensive search of the current literature, it is unlikely that we have missed any eligible studies. A limitation of our review is that we are unable to answer our research questions since we did not identify any eligible studies.

### 6.5 Agreements and disagreements with other studies or reviews

Currently, there is no primary study that has evaluated and compared the effectiveness of evidence‐informed practice to evidence‐based practice educational interventions. In addition, there is no previous systematic review that has compared the effectiveness of these two concepts. We are, therefore, unable to compare our results with other studies.

## 7 AUTHORS' CONCLUSIONS

### 7.1 Implications for practice

We are unable to make firm conclusions about the effectiveness of evidence‐informed practice versus evidence‐based practice educational interventions in improving undergraduate health and social care students' knowledge, attitudes, understanding, and behavior toward the application of evidence into practice.

Our review reveals extensive research work conducted on the effectiveness of evidence‐based practice educational interventions (see the Summary of findings table). Besides, several educational programs and interventions on evidence‐based practice have been developed for undergraduate health and social care students. However, there is limited evidence on evidence‐informed practice educational interventions and the effects of such interventions. Our finding reaffirms the importance attached to the evidence‐based practice concept and its use in decision‐making regarding health care practice and policy.

The purpose of evidence‐based practice to healthcare practice is to provide appropriate healthcare in a timely and effective manner to the patient (WHO, 2017). Evidence‐based practice is expected to improve patient outcomes, give job satisfaction, and provide cost‐effective care (Melnyk et al., 2010). Nevertheless, healthcare practitioners continue to struggle to implement the concept in clinical practice. Thus, there is an urgent need for a change in the way in which evidence is applied to healthcare practice. This change could be realized if other methods of applying evidence into practice, such as evidence‐informed practice, are considered and researched.

### 7.2 Implications for research

There is a need for primary studies evaluating the effectiveness of evidence‐informed practice versus evidence‐based practice educational interventions. Future research should reflect the differences and similarities between these two concepts in the educational interventions to be evaluated. In this way, the actual effect of each of the concepts could be determined and, compared with each other to determine the differences in the effect size.

Due to the limited literature on evidence‐informed practice interventions, future research in this area should be informed by a systematic map of the wider literature to determine models of evidence‐informed practice.

## CONTRIBUTIONS OF AUTHORS

- Content and Systematic Review methodology: Ms. Elizabeth Adjoa Kumah is a registered nurse who has worked mainly in the critical care setting as a nurse supervisor and patient advocate. She has been actively engaged in teaching healthcare students in the clinical setting and serving as a mentor. She has recently completed a Ph.D. Health program, with evidence‐informed practice and evidence‐based practice educational interventions as the area of research focus. She brings knowledge about the content both in terms of teaching healthcare students about the application of evidence into practice and theoretically for improving knowledge of evidence‐informed practice and how it enhances evidence‐based practice skills, attitude, and behavior in the educational setting. Elizabeth is passionate about improving the standard of patient care and patient outcome, which she believes could be achieved by effective and consistent implementation of evidence‐informed practice. She will also contribute to the methodological aspects of the systematic review.
- Content and Systematic review methods: Professor Robert McSherry will bring both methodological as well as content expertise relating to evidence‐informed practice and the development of teaching programmes to the team. His area of expertise is around evidence‐informed practice, patient safety, quality, and clinical governance using practice development. Practice development is about promoting person‐centered care and approaches, which Rob has integrated effectively within both educational and research programs. He is the co‐author of a book on systematic reviews and has over 30 years of experience as a registered nurse. Robs educational and professional expertise has been recognized and rewarded internationally and nationally. He was awarded the highly prestigious National Teaching Fellow award in the UK in 2011.
- Content and systematic review methods: Dr. Josette Bettany‐Saltikov will bring significant expertise of Systematic review methods and content to this systematic review, both in terms of knowledge about evidence‐based practice and knowledge about developing educational programs. She has taught systematic review methods to university students at all levels for over 15 years. She has also published a book on how to conduct a systematic review and has been involved in three Cochrane reviews, one of which she led. She has authored a number of systematic reviews on diverse topics published in other journals and has significant experience in developing educational programs from her teaching experience as a university Senior lecturer for 23 years.
- Content and systematic review methods: Professor Sharon Hamilton will bring expertise in systematic reviewing. She is the director of the Teesside Centre for Evidence‐Informed Practice: A Joanna Briggs Institute Centre of Excellence, and has conducted a number of qualitative and quantitative reviews. Sharon is a registered nurse and has research expertise in the evaluation of clinical interventions.
- Information retrieval: Mrs. Julie Hogg brings Information retrieval expertise to the team. Julie is an Academic Librarian at Teesside University and will carry out a thorough and systematic search of the literature.
- Statistical analysis: Mrs. Vicki Whittaker is a very experienced statistician with over 18 years of experience in teaching and advising students and academics on their research projects and clinical trials. She has been involved in data analysis and meta‐analysis of numerous research projects and systematic reviews.

## SUMMARY OF FINDINGS TABLES

Characteristics of excluded studies

**Table:** 

Study ID (lead author, year of publication)	Study title	Participants	Study design	Outcomes assessed	The country study was conducted	Reason for exclusion
1. Baarends, 2017	An exploratory study on the teaching of evidence‐based decision making	12 undergraduate occupational therapy students and their teacher	Explorative mixed‐methods study (semi‐structured interviews, questionnaires)	The effectiveness of evidence‐based decision making on the self‐efficacy and cognitive skills of undergraduate occupational therapy students	The Netherlands	The study does not compare evidence‐based practice educational interventions to evidence‐informed practice educational interventions.
2. Balakas, 2010	Teaching research and evidence‐based practice using a service‐learning approach	Undergraduate nursing students	Not clearly stated	The ability to use evidence in healthcare practice	Missouri, United States of America	The study does not compare evidence‐based practice to evidence‐informed practice educational interventions.
3. Boruff, 2011	Integrating evidence‐based practice and information literacy skills in teaching physical and occupational therapy students	First‐year physical and occupational therapy students	The authors designed an instructional activity that included workshops, lectures, and assignments that integrated evidence‐based practice and information literacy skills in a first‐year physical and occupational therapy program. Mode of delivery	Knowledge and skills in evidence‐based practice and information literacy	Canada	The study does not compare evidence‐based practice educational interventions to evidence‐informed practice educational interventions.
4. Brancato, 2006	An innovative clinical practicum to teach evidence‐based practice	Practicing Registered Nurses who are studying an undergraduate Bachelor of Science in Nursing program	Study design not stated.	Students ability to solve clinical problems using an evidence‐based practice approach	United States of America	The study does not compare evidence‐informed practice educational interventions to evidence‐based practice educational interventions.
			The authors used an innovative clinical intervention to enhance the student's ability to apply evidence‐based practice‐based.			Also, participants of the study were registered nurses.
5. Christie, 2012	How can we maximize nursing students' learning about research evidence and utilization in undergraduate, pre‐registration programs? A discussion paper	Not a primary study	Not a primary study	Not a primary study	Not a primary study	Not a primary paper
6. Cosme, 2018	Benchmarking of pre‐licensure nursing students' evidence‐based practice knowledge	57 Pre‐licensure nursing students	Quasi‐experimental study design	Evidence‐based practice knowledge of nursing students enrolled in a traditional Bachelor of Science in Nursing program	United States of America	The study does not compare evidence‐informed practice educational interventions to evidence‐based practice educational interventions.
						Also, participants of the study were registered nurses.
7. Davidson, 2016	Teaching evidence‐based practice using game‐based learning: improving the student experience	30 (after degree or second degree) undergraduate nursing students	Game platform analytics and thematic analysis of narrative comments in the midterms and end‐of‐course surveys were used to evaluate students' level of engagement. Student learning was evaluated using the end of a course letter grade.	Student experience in the evidence‐based practice undergraduate course. Student satisfaction, level of engagement, and overall achievement of learning outcomes	Canada	The study does not compare evidence‐informed practice educational interventions to evidence‐based practice educational interventions.
						Also, the outcomes measured did not meet the inclusion criteria.
8. Finotto, 2013	Teaching evidence‐based practice: developing a curriculum model to foster evidence‐based practice in undergraduate student nurses	300 newly graduated nurses	A descriptive study with the use of an anonymous questionnaire	Participants perception of Evidence‐based practice skills and competence	Italy	The study does not compare evidence‐informed practice educational interventions to evidence‐based practice educational interventions.
9. Finotto, 2010	Evidence‐based practice in nursing curricula: the experience of nursing degree course of Reggio Emilia. A pilot study	Full text not available in English (56 newly graduated nurses stated in the abstract)	Full text not available in English (study design stated in the abstract is Correlation‐descriptive)	Full text not available in English (students' perception of a 3‐year laboratory course on evidence‐based practice, to describe the laboratory course on evidence‐based practice, its objectives, its structure and its integration with practical training and nursing subjects)	Italy	The study does not compare evidence‐informed practice educational interventions to evidence‐based practice educational interventions. Also, the full text of the study is not available in English
10. Florin, 2011	Educational support for research utilization and capability beliefs regarding evidence‐based practice skills: a national survey of senior nursing students	2107 nursing students (from all 26 Swedish universities) in their last semester of undergraduate education.	Cross‐sectional survey design	Experience of educational support for research utilization, capability beliefs regarding evidence‐based practice skills, the relationship between educational support for research utilization, and capability beliefs regarding evidence‐based practice skills.	Sweden	Though the study determines the relationship between research utilization, which is a component of the evidence‐informed practice model (McSherry, 2007), and evidence‐based practice, it does not specifically compare evidence‐informed practice educational interventions to evidence‐based practice educational interventions.
11. Halcomb, 2009	Nursing student feedback on undergraduate research education: implications for teaching and learning	369 second‐year pre‐registration undergraduate nursing students	Mixed methods. Survey of students using a standardized tool utilized across the university to provide student feedback. The survey tool comprised of 13 items using a 5‐point Likert scale and two qualitative questions	To explore the challenges encountered when teaching an undergraduate research unit, to identify strategies that could be used to address these challenges in future programs	Australia	The study does not compare evidence‐informed practice educational interventions to evidence‐based practice educational interventions. Also, the outcomes measured did not meet the inclusion criteria.
12. Heye, 2009	Using new resources to teach evidence‐based practice	74 undergraduate nursing students	Development of an innovative strategy to teach evidence‐based practice. Participants did an oral and poster presentation as a form of feedback on the evidence‐based practice project	Student competencies for evidence‐based practice, faculty perceptions of the newly developed evidence‐based practice project	United States of America	The study does not compare evidence‐informed practice educational interventions to evidence‐based practice educational interventions.
						Also, the outcomes measured did not meet the inclusion criteria.
13. Hoffman, 2014	Brief training of student clinicians in shared decision making: a single‐blind randomized controlled trial	107 students. Students were either third‐year medical students, final‐year occupational therapy honors students, or postgraduate physiotherapy students undertaking a compulsory course in evidence‐based practice as part of their undergraduate or postgraduate degree. The medical students were enrolled in one university and the allied health students were in another university	A wait‐listed, multi‐center, single‐blind randomized controlled trial	Shared decision‐making skills, attitudes toward patient and clinician involvement in consultations, and confidence in communicating with patients about evidence.	Australia	Though the study focuses on shared decision making, which is a component of McSherry's (2007) evidence‐informed practice model, the study does not specifically compare evidence‐informed practice educational interventions to evidence‐based practice educational interventions.
14. Jang, 2015	The effect of an evidence‐based nursing course using action learning on undergraduate nursing students	Full‐text not in English	Full‐text not in English	Full‐text not in English	Full‐text not in English	Full‐text of the article is not in English. However, from the title of the study, it does not compare evidence‐informed practice educational interventions to evidence‐based practice educational interventions.
15. Janke, 2011	Promoting information literacy through collaborative service learning in an undergraduate research course	The third‐year undergraduate nursing students	The use of a collaborative service‐learning	Information literacy skills, students' appreciation of the role of evidence in nursing practice	Canada	The study does not compare evidence‐informed practice educational interventions to evidence‐based practice educational interventions.
16. Jorgensen, 2014	Does a 3‐week critical research appraisal course affect how students perceive their appraisal skills and the relevance of research for clinical practice? A repeated cross‐sectional survey	Final year undergraduate pre‐registered candidates in nursing, social work, child welfare, biochemistry, social education, and work and welfare.	Cross‐sectional survey design with a pre‐and post‐test	Students' attitudes toward using research and critical thinking	Norway	Though the study is focused on elements/terms (i.e., critical thinking, critical appraisal skills research utilization, the relevance of research for clinical practice), which are relevant to both evidence‐based practice and evidence‐informed practice, the study does not compare evidence‐informed practice educational interventions to evidence‐based practice educational interventions.
17. Katz, 2014	Skills in assessing the professional literature (SAPL): a 7‐year analysis of student evidence‐based practice performance	1647 dental students	Description of findings from an analysis of students' evidence‐based dentistry performance in assessing the professional literature	To describe student performance over a 7‐year period,	United States of America	The study does not compare evidence‐informed practice educational interventions to evidence‐based practice educational interventions.
18. Keib, 2017	Changes in nursing students' perception of research and evidence‐based practice after completing a research course	Third‐year undergraduate nursing students enrolled in required research and evidence‐based practice course.	A pre and post‐assessment design	Students' perception of and confidence in research and evidence‐based practice	United States of America	The study does not compare evidence‐informed practice educational interventions to evidence‐based practice educational interventions.
19. Kim, 2009	Evidence‐based practice‐focused interactive teaching strategy: a controlled study	208 4th‐year undergraduate nursing students studying at two nursing schools in the United States of America	A quasi‐experimental, controlled, pre‐and post‐test design	Knowledge, attitudes, use, and future use of evidence‐based practice	United States of America	The study does not compare evidence‐informed practice educational interventions to evidence‐based practice educational interventions.
20. Lawrence, 2012	Evidence‐based dental education: suggested course outlines for first‐and second‐year dental hygiene students	Not a primary study	Not a primary study	Not a primary study	Not a primary study	Not a primary study
21. Lauver, 2009	Toward evidence‐based teaching: evaluating the effectiveness of two teaching strategies in an associate degree nursing program	38 Associate of Science in Nursing (ASN) degree students	A quasi‐experimental design	To compare learning outcomes between two groups of students in an ASN degree program using two teaching methodologies: lecture and case instructions	United States of America	The study does not compare evidence‐informed practice educational interventions to evidence‐based practice educational interventions. Also, the outcomes measured do not meet the inclusion criteria
22. Leach, 2015	The impact of research education on student nurse attitude, skill and uptake of evidence‐based practice: a descriptive longitudinal survey	Third‐year nursing students enrolled in a Bachelor of Nursing program	Descriptive longitudinal survey	Attitudes, skills, and use of evidence‐based practice, barriers and facilitators of evidence‐based practice uptake	Australia	The study does not compare evidence‐informed practice educational interventions to evidence‐based practice educational interventions.
23. Leake, 2004	Teaming with students and a sacred cow contest to make changes in nursing practice	Second‐year nursing students	Not stated	Engaging nursing students and staff nurses in research by challenging established practices and exploring new ideas using the sacred cow contest	United States of America	The study does not compare evidence‐informed practice educational interventions to evidence‐based practice educational interventions. Also, the outcomes measured do not meet the inclusion criteria
24. Lewis, 2016	Diminishing effect sizes with repeated exposure to evidence‐based practice training in entry‐level health professional students: a longitudinal study	Entry‐level students in physiotherapy, podiatry, health science, medical radiations, and human movement.	An observational cross‐sectional analytic design	To explore the pattern of change in self‐reported and actual evidence‐based practice outcomes after one or two evidence‐based practice courses among entry‐level students and to consider the size of the change	Australia	The study does not compare evidence‐informed practice educational interventions to evidence‐based practice educational interventions. Also, the outcomes measured do not meet the inclusion criteria
25. Liou, 2013	Innovative strategies for teaching nursing research in Taiwan	Nursing students enrolled in a 2‐year registered nurse‐to ‐Bachelor of Science Nursing program	A descriptive, pretest‐posttest, quasi‐experimental design	Attitude toward research, classroom engagement, group learning, self‐directed learning, core professional abilities, perception of teaching strategies, and research knowledge.	Taiwan	The study does not compare evidence‐informed practice educational interventions to evidence‐based practice educational interventions. Also, study participants were registered nurses and so do not meet the inclusion criteria
26. Long, 2011	Entry‐level evidence‐based practice training in physiotherapy students: does it change knowledge, attitudes, and behavior? A longitudinal study	Entry‐level pre‐registered bachelor and master's physiotherapy students	A longitudinal pre‐post design	To explore self‐reported evidence‐based practice profiles (incorporating knowledge, attitudes, and behaviors) and actual evidence‐based practice knowledge of students following exposure to evidence‐based practice training courses.	Australia	The study does not compare evidence‐informed practice educational interventions to evidence‐based practice educational interventions.
27. Manns, 2015	A cross‐sectional study to examine evidence‐based practice skills and behaviors of physical therapy graduates: is there a knowledge‐to‐practice gap?	80 physical therapy graduates from 4 cohorts (i.e., 1996–2000, 2002–2005, 2005–2008, 2009–2010)	Cross‐sectional mixed‐methods study with 4 graduating cohorts	To examine differences in evidence‐based practice behaviors and knowledge among different cohorts of students trained in different curricula within the same university	Canada	The study does not compare evidence‐informed practice educational interventions to evidence‐based practice educational interventions.
28. Mary, 2014	Teaching evidence‐based practice and research through blended learning to undergraduate midwifery students from a practice‐based perspective	First‐year undergraduate midwifery students	The use of blended learning to teach evidence‐based practice	To describe the design, delivery, and evaluation of an undergraduate evidence‐based practice and research blended learning course	Australia	The study does not compare evidence‐informed practice educational interventions to evidence‐based practice educational interventions. Also, the outcomes measured do not meet the inclusion criteria
29. McCurry, 2009	Teaching undergraduate nursing research: a comparison of traditional and innovative approaches for success with millennial learners	72 junior baccalaureate nursing students	The use of a Likert scale to determine the effectiveness of a newly developed innovative educational intervention on evidence‐based practice versus traditional teaching methods of evidence‐based practice	To develop innovative strategies for teaching undergraduate nursing research that engages millennial learners and to compare students' perceived effectiveness of innovative strategies to traditional assignments	United States of America	The study does not compare evidence‐informed practice educational interventions to evidence‐based practice educational interventions. Also, the outcomes measured do not meet the inclusion criteria
30. McEvoy, 2018	Changes in physiotherapy students' knowledge and perceptions of evidence‐based practice from the first year to graduation: a mixed‐method	56 undergraduate physiotherapy students from the first year to graduation	Mixed methods with an explanatory sequential design	Self‐reported evidence‐based practice knowledge, attitudes and behaviors, and actual knowledge of evidence‐based practice	Australia	The study does not compare evidence‐informed practice educational interventions to evidence‐based practice educational interventions
31. Miller, 2003	Adapting an evidence‐based intervention: tales of the hustler project	1, 862 men	Not stated	Not relevant to this review	United States of America	The aims and objectives of the study are not relevant to this systematic review. The outcomes measure do not meet the inclusion criteria
32. Morris, 2016	The use of team‐based learning in a second‐year undergraduate pre‐registration nursing course on evidence‐informed decision making	Second‐year undergraduate pre‐registration nursing students	A post‐intervention evaluation involving; a cross‐sectional questionnaire survey, structured interviews with a convenience sample of 10 students, and student test results	Course organization using team‐based learning, perceptions of team‐based learning, and perceptions of team performance	United Kingdom	The study does not compare evidence‐informed practice educational interventions to evidence‐based practice educational interventions. Also, the outcomes measured do not meet the inclusion criteria
33. Ruzafa‐Martinez, 2016	Effectiveness of an Evidence‐Based Practice (EBP) course on the EBP competence of undergraduate nursing students: a quasi‐experimental study	Undergraduate nursing students enrolled in the second or third year of their nursing degree	A prospective, quasi‐experimental study was performed in a non‐randomized intervention group of nursing students who attended an evidence‐based practice course and a control group of nursing students who did not	Evidence‐based practice attitude, skills, and knowledge	Spain	The study does not compare evidence‐informed practice educational interventions to evidence‐based practice educational interventions.
34. Neville, 2008	Evidence‐based practice. Creating a spirit of inquiry to solve clinical nursing problems	10 professional nurses pursuing their Bachelor of Science in Nursing degrees	Case method approach	To explore the levels of evidence available in the conduct of evidence‐based literature search activities, to identify barriers in the conduct of evidence‐based practice, to gain an understanding of professional nurses' perception regarding the use of evidence‐based practice in clinical decision making.	United States of America	The study does not compare evidence‐informed practice educational interventions to evidence‐based practice educational interventions. Also, the outcomes measured, and study participants do not meet the inclusion criteria
35. Orta, 2016	Knowledge and competence of nursing faculty regarding evidence‐based practice	A convenience sample of 20 Registered Nurses‐to‐Bachelor of Science in Nursing faculty members.	A descriptive study of an online tutorial and resource center titled “introduction to evidence‐based practice: focusing on the must‐know”	Faculty members' knowledge of evidence‐based practice, faculty members' self‐confidence about their competency in evidence‐based practice	United States of America	The study does not compare evidence‐informed practice educational interventions to evidence‐based practice educational interventions. Also, the study participants do not meet the inclusion criteria
36. Raines, 2016	A collaborative strategy to bring evidence into practice	Second‐semester junior year students in a traditional Bachelor of Science in Nursing program	A collaborative teaching strategy was implemented with student nurses who were engaged in a 4‐weeks clinical rotation on a dedicated educational unit.	To evaluate students' understanding of the developed collaborative strategy and the quality of their work	United States of America	The study does not compare evidence‐informed practice educational interventions to evidence‐based practice educational interventions. Also, the outcomes measured do not meet the inclusion criteria
37. Santiago, 2018	Evidence‐based practice knowledge, attitude, access, and confidence: a comparison of dental hygiene and dental students	19 dental hygiene and 96 dental students	Pre‐ and post‐intervention survey	Evidence‐based practice knowledge, attitude, access, and confidence	United States of America	The study does not compare evidence‐informed practice educational interventions to evidence‐based practice educational interventions.
38. Scurlock‐Evans, 2017	To embed or not to embed: a longitudinal study exploring the impact of curriculum design on the evidence‐based practice profiles of UK pre‐registration nursing students	A convenience sample of 56 pre‐registration nursing students (55.4% studying an embedded evidence‐based practice curriculum and 44.6% studying a modular evidence‐based practice curriculum)	A longitudinal panel study design	Frequency of evidence‐based practice implementation, attitude toward evidence‐based practice, knowledge, and skills in retrieving and reviewing evidence, and knowledge and skills in applying and sharing evidence‐based practice	United Kingdom	The study does not compare evidence‐informed practice educational interventions to evidence‐based practice educational interventions.
39. Serfass, 2018	You're teaching evidence‐based practice to BSN students…But are they learning?	A convenience sample of nursing students nearing completion in a traditional Bachelor of Science in Nursing program was recruited from two campus sites of one nursing program	A multisite, cross‐sectional, descriptive study	Evidence‐based practice knowledge	United States of America	The study does not compare evidence‐informed practice educational interventions to evidence‐based practice educational interventions.
40. Smith‐Strom, 2012	Culture crush regarding nursing students' experience of implementation of evidence‐based practice in clinical practice	14 female undergraduate second‐year nursing students	A focus group method was adopted	To examine nursing students' experiences of the implementation of evidence‐based practice	Norway	The study does not compare evidence‐informed practice educational interventions to evidence‐based practice educational interventions. Also, the outcomes measured do not meet the inclusion criteria
41. Zelenikova, 2014	Perceptions of the effectiveness of evidence‐based practice courses by Czech nursing and midwifery students	119 nursing and midwifery students who were pursuing either a bachelor's or master's degree program	A descriptive cross‐sectional survey	Students' perception of the effectiveness of evidence‐based practice courses	Czech Republic	The study does not compare evidence‐informed practice educational interventions to evidence‐based practice educational interventions. Also, the outcomes measured do not meet the inclusion criteria
42. Cardoso, 2018	Evidence‐based practice educational program in nursing students' evidence‐based practice beliefs and knowledge, and the extent of their evidence‐based practice implementation	An ongoing study	An ongoing study	An ongoing study	An ongoing study in Portugal	An ongoing study. Also, the study does not compare evidence‐informed practice educational interventions to evidence‐based practice educational interventions.
43. Shorten, 2001	Developing information literacy: a key to evidence‐based nursing	Nursing students	The use of a curriculum‐integrated model through lectures and laboratory/tutorial sessions	To help nurses develop an awareness of the nursing literature, the skills to locate and retrieve it, and skills required in its evaluation	Australia	The study does not compare evidence‐informed practice educational interventions to evidence‐based practice educational interventions. Also, the outcomes measured do not meet the inclusion criteria
44. Jalali‐Nia, 2011	Effects of evidence‐based education on Iranian nursing students' knowledge and attitude	41 second‐year undergraduate nursing students studying two‐subject modules or medical‐surgical courses (musculoskeletal and gastrointestinal systems)	A quasi‐experimental post‐test design with a comparison group	Participants' knowledge of the principles of evidence‐based education, participants' knowledge about the subject matter (i.e., musculoskeletal and gastrointestinal systems), participants' attitudes toward evidence‐based education	Iran	The study does not compare evidence‐informed practice educational interventions to evidence‐based practice educational interventions.
45. Vetter, 2017	Tactics for teaching evidence‐based practice: enhancing active learning strategies with a large class of graduate EBP research in nursing students	58 students on a Master of Nursing degree program	The use of a 5‐point Likert scale to explore the student's perception about the effectiveness of the evidence‐based practice learning activity	To explore participants' perception about the effectiveness of the evidence‐based practice learning activity	United States of America	The study does not compare evidence‐informed practice educational interventions to evidence‐based practice educational interventions. Also, the outcomes measured do not meet the inclusion criteria
46. Bebermeyer, 2011	Teaching evidence‐based practice at the University of Texas Dental Branch at Houston	Not a primary research	Not a primary research	Not a primary research	Not a primary research	Not primary research.

## DECLARATIONS OF INTEREST

The review team declares no potential conflicts of interest.

## DIFFERENCES BETWEEN PROTOCOL AND REVIEW

In this review, we made every effort to identify eligible studies by following the methods outlined in the protocol.

In the protocol, we planned to assess whether evidence‐informed practice compared to evidence‐based practice educational interventions improve knowledge, attitudes, understanding, and behavior of undergraduate health and social care students toward the application of evidence into practice. In addition, we aimed to assess the impact of evidence‐informed practice and/or evidence‐based practice educational programs on patient outcomes. Examples of patient outcome indicators that we planned to assess include user experience, length of hospital stay, nosocomial infections, patient and health practitioner satisfaction, mortality, and morbidity rates. However, we could not explore these objectives in the final review because we did not identify any eligible studies for inclusion.

## PUBLISHED NOTES

### Characteristics of excluded studies

**Table:** 

Baarends et al., 2017	
**Reason for exclusion**	The study does not compare evidence‐based practice educational interventions to evidence‐informed practice educational interventions.
Balakas and Sparks, 2010	
**Reason for exclusion**	The study does not compare evidence‐based practice to evidence‐informed practice educational interventions.
Bebermeyer, 2011	
**Reason for exclusion**	Not a primary research
Boruff and Thomas, 2011	
**Reason for exclusion**	The study does not compare evidence‐based practice educational interventions to evidence‐informed practice educational interventions.
Brancato, 2006	
**Reason for exclusion**	The study does not compare evidence‐informed practice educational interventions to evidence‐based practice educational interventions.
Cardoso, 2018	
**Reason for exclusion**	This is an ongoing study. Also, the study does not compare evidence‐informed practice educational interventions to evidence‐based practice educational interventions.
Christie et al., 2012	
**Reason for exclusion**	Not a primary study.
Cosme et al., 2018	
**Reason for exclusion**	The study does not compare evidence‐informed practice educational interventions to evidence‐based practice educational interventions. Also, participants of the study were registered nurses.
Davidson and Candy, 2016	
**Reason for exclusion**	The study does not compare evidence‐informed practice educational interventions to evidence‐based practice educational interventions. Also, the outcomes measured did not meet the inclusion criteria.
Finotto et al., 2010	
**Reason for exclusion**	Study does not compare evidence‐informed practice educational interventions to evidence‐based practice educational interventions. Also, the full text of study is not available in English.
Finotto et al., 2013	
**Reason for exclusion**	The study does not compare evidence‐informed practice educational interventions to evidence‐based practice educational interventions.
Florin et al., 2011	
**Reason for exclusion**	Though the study determines the relationship between research utilization, which is a component of the evidence‐informed practice model (McSherry, 2007), and evidence‐based practice, it does not specifically compare evidence‐informed practice educational interventions to evidence‐based practice educational interventions.
Halcomb and Peters, 2009	
**Reason for exclusion**	Study does not compare evidence‐informed practice educational interventions to evidence‐based practice educational interventions. Also, the outcomes measured did not meet the inclusion criteria.
Heye and Stevens, 2009	
**Reason for exclusion**	The study does not compare evidence‐informed practice educational interventions to evidence‐based practice educational interventions. Also, the measured outcomes did not meet the inclusion criteria.
Hoffmann et al., 2014	
**Reason for exclusion**	Though study focusses on shared decision making, which is a component of McSherry's (2007) evidence‐informed practice model, the study does not specifically compare evidence‐informed practice educational interventions to evidence‐based practice educational interventions.
Jalali‐Nia et al., 2011	
**Reason for exclusion**	The study does not compare evidence‐informed practice educational interventions to evidence‐based practice educational interventions.
Jang et al., 2015	
**Reason for exclusion**	Full text of the article is not in English. However, from the title of the study, it does not compare evidence‐informed practice educational interventions to evidence‐based practice educational interventions.
Janke et al., 2012	
**Reason for exclusion**	The study does not compare evidence‐informed practice educational interventions to evidence‐based practice educational interventions.
Jelsness‐Jorgensen, 2015	
**Reason for exclusion**	Though the study is focused on elements/terms, which are relevant to both evidence‐based practice and evidence‐informed practice (i.e. critical thinking, critical appraisal skills research utilization, relevance of research for clinical practice), the study does not compare evidence‐informed practice educational interventions to evidence‐based practice educational interventions.
Katz et al., 2014	
**Reason for exclusion**	The study does not compare evidence‐informed practice educational interventions to evidence‐based practice educational interventions.
Keib et al., 2017	
**Reason for exclusion**	The study does not compare evidence‐informed practice educational interventions to evidence‐based practice educational interventions.
Kim et al., 2009	
**Reason for exclusion**	The study does not compare evidence‐informed practice educational interventions to evidence‐based practice educational interventions.
Laurence and Smith, 2014	
**Reason for exclusion**	Not a primary study
Lauver et al., 2009	
**Reason for exclusion**	The study does not compare evidence‐informed practice educational interventions to evidence‐based practice educational interventions. Also, the measured outcomes do not meet the inclusion criteria.
Leach et al., 2016	
**Reason for exclusion**	The study does not compare evidence‐informed practice educational interventions to evidence‐based practice educational interventions.
Leake, 2004	
**Reason for exclusion**	The study does not compare evidence‐informed practice educational interventions to evidence‐based practice educational interventions. Also, the outcomes measured do not meet the inclusion criteria.
Lewis et al., 2016	
**Reason for exclusion**	The study does not compare evidence‐informed practice educational interventions to evidence‐based practice educational interventions. Also, the outcomes measured do not meet the inclusion criteria.
Liou et al., 2013	
**Reason for exclusion**	The study does not compare evidence‐informed practice educational interventions to evidence‐based practice educational interventions. Also, study participants were registered nurses and so do not meet the inclusion criteria.
Long et al., 2011	
**Reason for exclusion**	The study does not compare evidence‐informed practice educational interventions to evidence‐based practice educational interventions.
Manns et al., 2015	
**Reason for exclusion**	The study does not compare evidence‐informed practice educational interventions to evidence‐based practice educational interventions.
Mary et al., 2014	
**Reason for exclusion**	The study does not compare evidence‐informed practice educational interventions to evidence‐based practice educational interventions. Also, the outcomes measured do not meet the inclusion criteria.
McCurry and Martins, 2010	
**Reason for exclusion**	The study does not compare evidence‐informed practice educational interventions to evidence‐based practice educational interventions. Also, the outcomes measured do not meet the inclusion criteria.
McEvoy et al., 2018	
**Reason for exclusion**	The study does not compare evidence‐informed practice educational interventions to evidence‐based practice educational interventions.
Miller, 2003	
**Reason for exclusion**	The aims and objectives of the study are not relevant to this systematic review. The outcomes measured do not meet the inclusion criteria.
Morris, 2016	
**Reason for exclusion**	The study does not compare evidence‐informed practice educational interventions to evidence‐based practice educational interventions. Also, the outcomes measured do not meet the inclusion criteria.
Neville and Horbatt, 2008	
**Reason for exclusion**	The study does not compare evidence‐informed practice educational interventions to evidence‐based practice educational interventions. Also, the outcomes measured, and the study participants do not meet the inclusion criteria.
Orta et al., 2016	
**Reason for exclusion**	The study does not compare evidence‐informed practice educational interventions to evidence‐based practice educational interventions. Also, the study participants do not meet the inclusion criteria.
Raines, 2016	
**Reason for exclusion**	The study does not compare evidence‐informed practice educational interventions to evidence‐based practice educational interventions. Also, the outcomes measured do not meet the inclusion criteria.
Ruzafa‐Martinez et al., 2016	
**Reason for exclusion**	The study does not compare evidence‐informed practice educational interventions to evidence‐based practice educational interventions.
Santiago et al., 2018	
**Reason for exclusion**	The study does not compare evidence‐informed practice educational interventions to evidence‐based practice educational interventions.
Scurlock‐Evans et al., 2017	
**Reason for exclusion**	The study does not compare evidence‐informed practice educational interventions to evidence‐based practice educational interventions.
Serfass and Hagedorn Wonder, 2018	
**Reason for exclusion**	The study does not compare evidence‐informed practice educational interventions to evidence‐based practice educational interventions.
Shorten et al., 2001	
**Reason for exclusion**	The study does not compare evidence‐informed practice educational interventions to evidence‐based practice educational interventions. Also, the outcomes measured do not meet the inclusion criteria.
Smith‐Strom et al., 2012	
**Reason for exclusion**	The study does not compare evidence‐informed practice educational interventions to evidence‐based practice educational interventions. Also, the outcomes measured do not meet the inclusion criteria.
Vetter and Latimer, 2017	
**Reason for exclusion**	The study does not compare evidence‐informed practice educational interventions to evidence‐based practice educational interventions. Also, the outcomes measured do not meet the inclusion criteria.
Zeleníková and Jarošová, 2014	
**Reason for exclusion**	The study does not compare evidence‐informed practice educational interventions to evidence‐based practice educational interventions. Also, the outcomes measured do not meet the inclusion criteria.

## SOURCES OF SUPPORT

Internal sources

- Teesside University, UK

This review forms part of a Ph.D programme, which is supported and funded by the Teesside University, Middlesbrough.

External sources

- Not applicable to the current systematic review, UK

This systematic review did not receive any form of external support.

## Supporting information

Supporting information.
