# Supplementary material for: Evidence‐informed practice versus evidence‐based practice educational interventions for improving knowledge, attitudes, understanding, and behavior toward the application of evidence into practice: A comprehensive systematic review of UG student
Source: Campbell Syst Rev. 2022 Apr 16;18(2):e1233. doi: 10.1002/cl2.1233 (PMC9013402; doi:10.1002/cl2.1233)
Supplement: Supplementary file 1 — Supporting information. [file CL2-18-e1233-s001.docx]

# Appendices

# Appendices

## 1 MEDLINE Search Strategy

| # | Query |
| --- | --- |
| S76 | S17 AND S35 AND S51 AND S75 |
| S75 | S52 OR S53 OR S54 OR S55 OR S56 OR S57 OR S58 OR S59 OR S60 OR S61 OR S62 OR S63 OR S64 OR S65 OR S66 OR S67 OR S68 OR S69 OR S70 OR S71 OR S72 OR S73 OR S74 |
| S74 | "mixed method*" |
| S73 | trial* |
| S72 | "epidemiological stud*" |
| S71 | "before and after stud*" |
| S70 | "retrospective stud*" |
| S69 | "prospective stud*" |
| S68 | "descriptive stud*" |
| S67 | "grounded theory" |
| S66 | phenomenolog* |
| S65 | interview* |
| S64 | "focus group" |
| S63 | "cross sectional" |
| S62 | "case series" |
| S61 | "cohort stud*" |
| S60 | ethnography |
| S59 | "qualitative stud*" |
| S58 | experiment* |
| S57 | "trial registration" |
| S56 | "control group design" |
| S55 | "control* trial*" |
| S54 | "control* design*" |
| S53 | randomi?ed control* trial* or rtc* or random* control* trial* |
| S52 | random* |
| S51 | S36 OR S37 OR S38 OR S39 OR S40 OR S41 OR S42 OR S43 OR S44 OR S45 OR S46 OR S47 OR S48 OR S49 OR S50 |
| S50 | "patient experience*" |
| S49 | "quality of care" |
| S48 | "patient outcome*" |
| S47 | "professional responsibilit*" |
| S46 | "professional accountability" |
| S45 | "informed decision-making" |
| S44 | "research implement*" |
| S43 | "research aware*" |
| S42 | "application of evidence-informed*" |
| S41 | "application of evidence-based*" |
| S40 | "application of knowledge*" |
| S39 | understand* |
| S38 | behavio* |
| S37 | attitude* |
| S36 | knowledge* |
| S35 | S18 OR S19 OR S20 OR S21 OR S22 OR S23 OR S24 OR S25 OR S26 OR S27 OR S28 OR S29 OR S30 OR S31 OR S32 OR S33 OR S34 |
| S34 | lecture* |
| S33 | course* |
| S32 | "education* program*" |
| S31 | "education* intervention" |
| S30 | evidence-based practice vurses evidence-informed practice |
| S29 | compar* evidence-based practice and evidence-informed practice |
| S28 | "evidence-informed practice teaching and learning" |
| S27 | "evidence-informed practice teaching and learning" |
| S26 | "evidence-based practice teaching and learning" |
| S25 | "evidence-informed practice education" |
| S24 | "evidence-based practice education" |
| S23 | ebp |
| S22 | eip |
| S21 | evidence-informed* |
| S20 | evidence-based* |
| S19 | "evidence-informed practice" |
| S18 | "evidence-based practice" |
| S17 | S1 OR S2 OR S3 OR S4 OR S5 OR S6 OR S7 OR S8 OR S9 OR S10 OR S11 OR S12 OR S13 OR S14 OR S15 OR S16 |
| S16 | undergraduate student* |
| S15 | baccalaureate |
| S14 | undergraduate social care student* |
| S13 | undergraduate healthcare student* |
| S12 | dental therap* student* |
| S11 | dental hygiene student* |
| S10 | (MH "Students, Dental") |
| S9 | paramedic student* |
| S8 | social work student* |
| S7 | radiography student* |
| S6 | student* midwi* |
| S5 | occupation* therap* student* |
| S4 | (MH "Students, Health Occupations") |
| S3 | student* physical therap* |
| S2 | student* physiotherap* |
| S1 | (MH "Student* Nurs*") |

## 2 Cochrane Data Collection form for intervention reviews for RCTs and non-RCTs

<https://dplp.cochrane.org/data-extraction-forms>
